# Supplementary material for: Randomised feasibility trial of the helping families programme-modified: an intensive parenting intervention for parents affected by severe personality difficulties
Source: BMJ Open. 2020 Feb 6;10(2):e033637. doi: 10.1136/bmjopen-2019-033637 (PMC7045220; doi:10.1136/bmjopen-2019-033637)
Supplement: Author's manuscript [file bmjopen-2019-033637.draft_revisions.pdf]

BMJ Open

BMJ Open is committed to open peer review. As part of this commitment we make the peer review history of every article we publish publicly available.

When an article is published we post the peer reviewers' comments and the authors' responses online. We also post the versions of the paper that were used during peer review. These are the versions that the peer review comments apply to.

The versions of the paper that follow are the versions that were submitted during the peer review process. They are not the versions of record or the final published versions. They should not be cited or distributed as the published version of this manuscript.

BMJ Open is an open access journal and the full, final, typeset and author-corrected version of record of the manuscript is available on our site with no access controls, subscription charges or pay-per-view fees (http://bmjopen.bmj.com).

If you have any questions on BMJ Open's open peer review process please email
info.bmjopen@bmj.com

BMJ Open

Randomised Feasibility Trial of the Helping Families Programme-Modified: An Intensive Parenting Intervention for Parents Affected by Severe Personality Difficulties

Journal:	*BMJ Open*
Manuscript ID	bmjopen-2019-033637
Article Type:	Original research
Date Submitted by the Author:	20-Aug-2019

Complete List of Authors:	Day, Crispin; King's College London, ; South London and Maudsley NHS Foundation Trust,<br>Briskman, Jackie; King's College London<br>Crawford, Mike; Imperial College London, Centre for Psychiatry<br>Foote, Lisa; McPin Foundation<br>Harris, Lucy; South London and Maudsley NHS Foundation Trust,<br>Boadu, Janet; King's College London<br>McCrone, Paul; King's College London,<br>McMurran, Mary; University of Nottingham<br>Michelson, Daniel ; University of Sussex, School of Psychology<br>Moran, Paul; Univiersity of Bristol, Centre of Academic Mental Health, School of Social and Community Medicine; University of Bristol<br>Mosse, Liberty; King's College London<br>Scott, Stephen; King's College London<br>Stahl, Daniel; Department of Biostatistics Institute of Psychiatry, Psychology & Neuroscience (IoPPN), King's College London<br>Ramchandani, Paul; University of Cambridge<br>Weaver, Tim ; Middlesex University

Keywords:	Personality disorders < PSYCHIATRY, Parenting, Child behaviour, MENTAL HEALTH

SCHOLARONE™
Manuscripts

I, the Submitting Author has the right to grant and does grant on behalf of all authors of the Work (as defined in the below author licence), an exclusive licence and/or a non-exclusive licence for contributions from authors who are: i) UK Crown employees; ii) where BMJ has agreed a CC-BY licence shall apply, and/or iii) in accordance with the terms applicable for US Federal Government officers or employees acting as part of their official duties; on a worldwide, perpetual, irrevocable, royalty-free basis to BMJ Publishing Group Ltd ("BMJ") its licensees and where the relevant Journal is co-owned by BMJ to the co-owners of the Journal, to publish the Work in this journal and any other BMJ products and to exploit all rights, as set out in our licence.

The Submitting Author accepts and understands that any supply made under these terms is made by BMJ to the Submitting Author unless you are acting as an employee on behalf of your employer or a postgraduate student of an affiliated institution which is paying any applicable article publishing charge ("APC") for Open Access articles. Where the Submitting Author wishes to make the Work available on an Open Access basis (and intends to pay the relevant APC), the terms of reuse of such Open Access shall be governed by a Creative Commons licence – details of these licences and which Creative Commons licence will apply to this Work are set out in our licence referred to above.

Other than as permitted in any relevant BMJ Author's Self Archiving Policies, I confirm this Work has not been accepted for publication elsewhere, is not being considered for publication elsewhere and does not duplicate material already published. I confirm all authors consent to publication of this Work and authorise the granting of this licence.

For peer review only

Randomised Feasibility Trial of the Helping Families Programme-Modified: An Intensive Parenting Intervention for Parents Affected by Severe Personality Difficulties

Crispin Day*[a], Jackie Briskman[b], Mike J Crawford[c], Lisa Foote[d], Lucy Harris[a], Janet Boadu[e], Paul McCrone[e], Mary McMurran[f], Daniel Michelson[g], Paul Moran[h], Liberty Mosse[a], Stephen Scott[i], Daniel Stahl[j], Paul Ramchandani[k], & Timothy Weaver[l].

*Corresponding author

[a] CAMHS Research Unit, Centre for Parent and Child Support, South London and Maudsley NHS Foundation Trust, Michael Rutter Centre, De Crespigny Park, London SE5 8AF, UK. Email: crispin.1.day@kcl.ac.uk

[b] Dept. of Psychology, Institute of Psychiatry, Psychology and Neuroscience, King's College London, De Crespigny Park, London SE5 8AF, UK.

[c] The Centre for Psychiatry, Imperial College London, 7th Floor Commonwealth Building, Hammersmith Hospital Campus, London W12 0NN, UK.

[d] McPin Foundation, 32-36 Loman St, London SE1 0EH, UK.

[e] King's Health Economics, P024 David Goldberg Centre, Institute of Psychiatry, Psychology & Neuroscience, King's College London, De Crespigny Park, London SE5 8AF, UK.

[f] Institute of Mental Health, University of Nottingham, Triumph Road, Nottingham, NG7 2TU, UK.

[g] School of Psychology, University of Sussex, Falmer, Brighton BN1 9QH

[h] Centre for Academic Mental Health, Department of Population Health Sciences, Bristol Medical School, University of Bristol, Bristol BS8 2BN, UK.

[i] Department of Child and Adolescent Psychiatry, Institute of Psychiatry, Psychology and Neuroscience, King's College London, De Crespigny Park, London SE5 8AF, UK.

[j] Department of Biostatistics, Institute of Psychiatry, Psychology and Neuroscience, King's College London, De Crespigny Park, London SE5 8AF, UK.

[k] PEDAL Research Centre, Faculty of Education, University of Cambridge, UK.

[l] Department of Mental Health, Social Work and Integrative Medicine, School of Health and Education, Middlesex University, The Burroughs, London NW4 4BT, UK.

For peer review only

Randomised Feasibility Trial of the Helping Families Programme-Modified: An Intensive Parenting Intervention for Parents Affected by Severe Personality Difficulties

Abstract

Background

Specialist parenting intervention could improve coexistent mental health problems of parents affected by severe personality difficulties and their children.

Objective

Conduct a feasibility trial of Helping Families Programme-Modified (HFP-M), a specialist parenting intervention.

Design

Pragmatic, mixed-methods trial, 1:1 random allocation, assessing feasibility, intervention acceptability and outcome estimates.

Settings

Two UK NHS health trusts and concomitant local authority children's social care services

Participants

Parents: (i) primary caregiver; (ii) 18-65 years; (iii) severe personality difficulties, (iv) proficient English, and (v) capacity for consent. Child: (i) aged 3-11 years; (ii) living with index parent, (iii) significant emotional/behavioural difficulties.

Intervention

HFP-M: specialist 16-session home-based intervention using structured, goal-orientated parenting and therapeutic engagement strategies. Usual care: standard care augmented by a single psychoeducational parenting session.

Outcomes

Feasibility parameters: rates of recruitment, eligibility, allocation, retention, data completion and experience. Intervention acceptability: rates of acceptance, completion, and alliance

For peer review only

(Working Alliance Inventory-Short Revised). Outcomes: child (Eyberg Child Behavior Inventory, Concerns About My Child, Child Behavior Checklist-Internalising Scale), parenting (Arnold-O'Leary Parenting Scale, Kansas Parental Satisfaction Scale, and parent mental health (Symptom Checklist-27. Researchers collecting quantitative data were blind to allocation status.

Results

Findings broadly supported trial feasibility using non-diagnostic selection criterion. Of 48 participants recruited, 32 completed post intervention measures at mean 42 weeks later. Post-intervention retention exceeded *a priori* rate (HFP-M=18; Usual care=14; 66.7%, 95% C.I. 51.6%-79.6%;). HFP-M was acceptable, with delivery longer than planned. Usual care condition had lower alliance rating. Child and parenting outcome effects detected across trial arms with potential HFP-M advantage (ES range: 0.0-1.3).

Conclusion

HFP-M is an acceptable and potentially effective specialist parenting. A definitive trial is feasible, subject to consideration of recruitment and retention methods, intervention efficiency and comparator condition. Caution is required in interpretation of results due to reduced sample size. No serious adverse events reported.

Trial registration

ISRCTN14573230

For peer review only - http://bmjopen.bmj.com/site/about/guidelines.xhtml

Article Summary

Strengths and Limitations of this Study

- This is the first feasibility trial of a specialist parenting intervention for coexistent mental health problems of parents affected by severe personality difficulties and their children.
- Findings support further research to test the specialist parenting intervention in a definitive trial, with modifications required to improve intervention efficiency and participant retention.
- Caution is required in interpretation of results due to reduced sample size.
- The trial population's complex personality difficulties underline the importance of effective and sensitive management of trial consent procedures, random allocation and ongoing engagement of participants, particularly for those allocated to the usual care condition.

Keywords

Child behaviour, parenting, mental health, family relations

Funding

This work was supported by National Institute of Health Research, Health Technology Assessment, Project Reference Number: 12/194/01.

For peer review only

Contributions of the Authors

Dr Crispin Day, Head, Centre for Parent and Child Support, South London and Maudsley NHS Foundation Trust and Head, CAMHS Research Unit, King's College, London, was the chief investigator and responsible for the overall conception, design, data acquisition, analysis, interpretation of findings at each phase of this research. He is responsible for the overall content of this report.

Ms Jackie Briskman, Department of Psychology, King's College, London, was the senior research trial coordinator and was responsible for data acquisition, analysis, interpretation, drafting findings and revising this report.

Professor Mike J. Crawford, Professor in Mental Health Research, Centre for Psychiatry, Imperial College, London was a co-applicant and contributed to the overall conception, design and interpretation of findings at each phase of this research. He provided an expert contribution about personality disorder. He contributed to critically revising this report.

Ms Lisa Foote, McPin Foundation, was a service user researcher and representative in this research. She contributed to the design, data acquisition, analysis, interpretation and drafting of findings. She contributed to critically revising this report

Dr Lucy Harris, Deputy Head, Centre for Parent and Child Support, South London and Maudsley NHS Foundation Trust, was responsible for the development and revision of Helping Families Programme-Modified and supervised trial therapists. She contributed to critically revising this report.

Ms Janet Boadu is a researcher at King's Health Economics, King's College, London, co-conducted the health economic analysis and drafted findings for this report.

Professor Paul McCrone, Professor of Health Economics and Director of King's Health Economics, King's College, London, was a co-applicant and contributed to the overall conception, design and interpretation of findings at each phase of this research. He led the health economic component of the research and contributed to critically revising this report.

Professor Mary McMurran, Professor at the Institute of Mental Health, University of Nottingham was a co-applicant and contributed to the overall conception, design and interpretation of findings at each phase of this research. She provided an expert contribution about personality disorder. She was a member of the Manualisation Working Group, contributed to research therapist raining and contributed to critically revising this report.

For peer review only - http://bmjopen.bmj.com/site/about/guidelines.xhtml

Dr Daniel Michelson, Senior Lecturer in Clinical Psychology, School of Psychology, University of Sussex, was a co-applicant and worked closely with the chief investigator on the overall conception and design of this research. He was senior research coordinator during the intervention development phase leading up to this trial and contributed to critically revising this report.

Professor Paul Moran, Professor of Psychiatry, Population Health Sciences, University of Bristol, was a co-applicant and contributed to the overall conception, design and interpretation of findings at each phase of this research. He provided an expert contribution about personality disorder. He contributed to research therapist raining and critically revised this report.

Ms Liberty Mosse, CAMHS Research Unit, King's College, London, was a researcher worker on this study. She was responsible for Phase 3 data acquisition, analysis and interpretation of findings. She completed the majority of qualitative data acquisition and led its analysis under the direction of the chief investigator. She contributed to critically revising this report

Professor Stephen Scott, Professor of Child Health and Behaviour, King's College, London, was a co-applicant and contributed to the overall conception, design and interpretation of findings at each phase of this research. He provided an expert contribution about parenting and parenting programmes. He contributed to critically revising this report.

Dr Daniel Stahl, Reader in Biostatistics, Biostatistics and Health Informatics, King's College, London, was a co-applicant and contributed to the overall conception, design and interpretation of findings at each phase of this research. He provided an expert contribution to the design, analysis and interpretation of quantitative components of the research. He contributed to critically revising this report.

Professor Paul Ramchandani, LEGO Professor of Play in Education, Development and Learning, Faculty of Education, University of Cambridge, was a co-applicant and contributed to the overall conception, design and interpretation of findings at each phase of this research. He provided an expert contribution about child mental health and co-ordinated the research in CNWL. He contributed to critically revising this report.

Dr Timothy Weaver, Associate Professor in Mental Health, Mental Health Social Work & Interprofessional Learning, Middlesex University, London, was a co-applicant and

For peer review only

contributed to the overall conception, design and interpretation of findings at each phase of this research. He provided an expert contribution about qualitative design, data acquisition and interpretation. He contributed to critically revising this report.

Manual and Data Availability

All manuals can be obtained from the corresponding author. All data requests should be submitted to the corresponding author for consideration. Access to anonymised data may be granted following review.

Acknowledgements

We are indebted to the parents, practitioners, clinicians and service managers who worked with us on this trial, particularly Ruth Wilson. We would particularly like to thank Sarah Inkpen, Bethan Stevenson and Chelsea McCorry for their help with trial findings. Peter Fonagy and Philip Graham for overseeing the governance of the trial

For peer review only

Introduction

Mental ill health is the largest cause of disability, with three-quarters of lifetime disorders starting during childhood.[1] Children of parents affected by severe personality difficulties are at particular risk due to the impact of parents' symptoms and associated impairment on parenting capacity.[2-4] Lack of evidence-based treatments, under-developed care pathways and stigma result in poorer immediate and longer-term outcomes for children, increase likelihood of intergenerational transmission of mental health difficulties and perpetuate social disadvantage.[5,6]

Severe personality difficulties, including personality disorders, affect over 4% of UK adults and 40% of mental health service users, at least one-quarter of whom are parents. [7,8] Per annum UK treatment costs exceed £70 million with wider societal costs estimated at £8 billion per year. [9] Characterised by highly problematic interpersonal, family and social relationships, emotional dysregulation, and poor impulse control, severe parental personality difficulties are associated with insensitive and intrusive interactions with offspring, family hostility, inconsistent and unpredictable family routines that undermine affectionate, stable, and responsive parenting required for healthy child development.[2-4] Affected parents are likely to suffer higher levels of parenting stress and lower satisfaction, exacerbating their own mental health difficulties.[10,11]

One in ten UK children suffer mental health disorders, with children of parents with severe personality difficulties at substantially higher risk, up to 70%, of intergenerational transmission, most commonly behavioural disorders.[12,13] Mental health disorders during childhood are associated with academic failure, school exclusion, maltreatment, self-harm and gang affiliation, with longer term life-time risks of comorbid mental and physical health conditions, drug misuse, offending and worklessness.[1,12,13] Annual UK public service costs for severe behavioural problems are estimated at £5,000 per child including £1400 health costs. UK lifetime estimated costs per case range from £85,000 (moderate case) to £260,000 (severe case).[14,15]

The development and implementation of effective care models for co-occurring child and parental mental health problems are significant health policy and research priorities.[16-19] Nevertheless, routine care is highly variable and fragmented, generally focused on the needs of *either* the adult *or* the child,[17] resulting in problem under-identification, poor understanding of the interrelationship between child and parent difficulties, and

For peer review only

For peer review only - http://bmjopen.bmj.com/site/about/guidelines.xhtml

misattribution of parenting difficulties to adult mental health symptoms *per se*.[20] Affected parents can be reluctant to engage in interventions due to associated stigma, treatment scepticism, fears about child protection proceedings, and the interpersonal difficulties and adverse life circumstances associated with personality difficulties.[21]

Parenting and parent factors are central to much effective child mental health treatment and problem remediation. Parenting programmes are the recommended cost-effective treatment for child behaviour problems.[22,23] However, non-specialised parenting programmes, not specifically designed for parents affected by mental health difficulties, often result in poorer engagement, acceptability and outcomes for this population.[24,25] Specialist parenting interventions for some parental mental health conditions, such as depression, substance misuse and eating disorders, have demonstrated improved outcomes and reductions in intergenerational transmission of mental illness, by up to 40%.[26,27] Specialist programmes for parents affected by personality difficulties are at an earlier stage of genesis and evidence production.

The Helping Families Programme-Modified (HFP-M) was developed as a specialist, intensive parenting interventions to address this need and service gap. Its aim is to improve immediate child and parenting outcomes with longer-term potential to reduce intergenerational transmission and psychosocial adversity within affected families.[4] HFP-M development was guided by recommended frameworks, involving synthesis of two existing evidence-based interventions, integration of relevant clinical practice recommendations and service user consultation.[22,28-33] HFP-M's structured approach has three core components to achieve its aims [34]: (i) Core Therapeutic Process: including partnership and goal-based methods intended to promote collaborative engagement and shared formulation, empathic parent validation, and crisis management;[34] (ii) Parent Groundwork: including emotion-focussed, cognitive, behavioural and interpersonal strategies intended to mitigate the effects of parental emotional dysregulation and hostility on parenting and family function, and (iii) Parenting Strategies: including skills development and use of positive parenting methods, attitudes, emotional care and reflective function

Definitive trials require successful participant recruitment and retention, and intervention acceptability.[30, 35-39] Participant retention underpins trial validity, with lower rates reducing power, undermining interpretation of findings and increasing costs. HFP-M participant retention, and initial recruitment, was expected to be affected by: (i) participants'

For peer review only - http://bmjopen.bmj.com/site/about/guidelines.xhtml

core clinical features, (ii) their greater exposure to family stress, negative life events, lower levels of social support and co-morbid mental health conditions, and (iii) under-identification of need within routine care, negative referrer expectancies, lower service engagement and non-attendance within clinical services. The impact of these factors is reflected in evidence derived from 45 personality disorder treatments trials reported in two systematic reviews indicating a median participant non-completion rate of 35%.[40,41] Previous field testing had indicated that trial recruitment based on research diagnosis of personality disorder was unlikely to be viable.[21]

To be useful and effective in practice, interventions need to demonstrate both clinical efficacy and user acceptability. Acceptability refers to service user judgements about an intervention across four inter-related domains of satisfaction: (i) intervention relevance, (ii) intervention content and procedures, (iii) clinician/provider characteristics and (iv) outcome suitability.[37,38]

This study reports quantitative findings from a randomised feasibility trial of HFP-M, the protocol for which has been published [4]. The aim was to assess the feasibility of research procedures and intervention delivery with findings being used to inform the design of a full-scale trial. More specifically, the trial sought to obtain evidence for: (i) rates of participant identification, recruitment and retention, the primary *a priori* feasibility criterion was a retention rate of at least 65% post-intervention, (ii) intervention acceptability, content and fidelity, and (iii) effect sizes and variance estimates for child and parent outcomes necessary to power a full-scale trial, with child behaviour nominated as the primary clinical outcome in the feasibility trial. Qualitative findings from a parallel process evaluation investigating the influence of contextual factors on intervention implementation and outcome generation are published elsewhere, as are preliminary intervention costs and estimates of cost-effectiveness.[42]

Method

Design

Mixed-method, two-arm, parallel feasibility trial with random allocation of consenting parents in a 1:1 ratio to either (i) HFP-M or (ii) Usual care.

For peer review only - http://bmjopen.bmj.com/site/about/guidelines.xhtml

Quantitative data were intended to be collected at pre-randomisation baseline (Time 1), post-intervention (Time 2), six months from baseline; and follow-up (Time 3), 4-month, ten months from baseline.

Eligibility criteria

Parent: (i) primary parental caregiver for index child; (ii) aged 18-65 years; (iii) experiencing severe personality difficulties, assessed by self-administered Standardised Assessment of Personality - Abbreviated Scale (SAPAS) score of ≥ 3 [43], (iv) proficient written and spoken English, and (v) capacity to provide informed consent.

Child: (i) aged 3-11 years; (ii) living with index parent; (iii) experiencing significant emotional and/or behavioural difficulties, assessed by Strengths and Difficulties Questionnaire [44] Total Score of ≥17.

Exclusion criteria: Parent (i) co-existing psychosis; (ii) engagement in another structured parenting intervention; (iii) mental health inpatient status or (iv) insufficient language/cognitive abilities; Child (i) pervasive developmental disorder; (ii) not residing with index parent; and (iii) considered for/subject to child protection proceedings/supervision.

Interventions

HFP-M Intervention

16 session home-based one-one parenting intervention for parents with severe personality difficulties, including personality disorder. Session modules proceeded iteratively to (i) establish an effective, validating collaborative partnership, (ii) explore and develop shared understanding of the impact of severe parental personality difficulties on parenting, child functioning and wider family ecology, (iv) implement parent quick wins and focal parenting and child intervention goals, (v) use of evidence-based parenting and parent self-care strategies to achieve change and agreed goals, and (vi) recurrent review of goal attainment and therapeutic partnership. Six trial therapists delivered HFP-M, receiving eight, three-hour, training sessions provided by HFP-M programme developers and relevant clinical experts followed by fortnightly supervision to support clinical implementation and fidelity.

Usual care

No systematised parenting pathway was typically provided for the participant population. (REF). To provide consistent and low intensity support, participants could choose to receive a single one-to-one parent information and support session in addition to their existing care.

For peer review only - http://bmjopen.bmj.com/site/about/guidelines.xhtml

Derived from the evidence-based Empowering Parents Empowering Communities parenting programme, [45] session content included: (i) brief exploration of parenting and child needs, family support, parent priorities and goals, and (ii) focus on one parent priority topic, selected from Being Good Enough, Listening to My Child, Praising My Child, Taking Care of Myself, Understanding My Child's Behaviour, My Child's Emotion or Playing Together. The additional session was delivered by three trained parent practitioners, who received ongoing supervision to support implementation and fidelity, including module content delivery.

Concomitant interventions

Trial interventions were delivered in conjunction with existing medical, psychosocial and educational support and treatment services used by participating parents and their families.

Measures

Participant characteristics

Descriptive data were collected on parent and child age, gender and ethnicity, family household composition, participant diagnostic, status, and family socio-economic status. Data from EQ-5D, [46] a standardised measure of health status developed by EuroQol Group, provided information about parent and child health and disability.

Feasibility evaluation

Structured record sheets, completed prospectively by research staff and trial therapists, documented (i) participant identification and verbal consent, (ii) screening, eligibility, informed written consent, randomisation, and reasons for non-participation, and (iii) data collection, including reasons for missing data.

Clinical outcomes

- **Eyberg Child Behavior Inventory (ECBI)**,[47] a 36-item questionnaire assessing intensity and number of disruptive behaviour problems in 2-16 year-olds, providing a comprehensive measure of child behaviour difficulties. Intensity Scale score of $\geq$ 131 indicates significant severity. Problem Scale score of $\geq$ 15 indicates significant number of problems.
- **Concerns About My Child (CAMC)**,[45] a visual analogue scale (0-100) rating three parental concerns about their child. Concerns nominated at baseline were re-rated at each time point, providing a sensitive, individualised index of change.

For peer review only

- **Child Behavior Checklist-Internalising Scale (CBCL-Int)**,[48] a 32-item questionnaire assessing internalising problems in 6-18 year-olds, with an alternate 36-item version for children aged 1½-5 years. Standardised T-scores combine results from both versions. A score of ≥60 indicates clinical caseness.
- **Arnold-O'Leary Parenting Scale (PS)**,[49] a 30-item questionnaire assessing dysfunctional parental discipline behaviour for children aged 2-16 years, which correlates with more time-consuming observational ratings. A score of ≥ 3.2 differentiates clinic and non-referred children.
- **Kansas Parental Satisfaction Scale (KPSS)**,[50] a 3-item scale providing a brief measure of parenting stress and satisfaction.
- **Symptom Checklist-27 (SCL-27)**,[51] a 27-item questionnaire assessing psychological symptoms in adults that provides a Global Severity Index (GSI) of psychopathology.

Intervention acceptability:

- **Working Alliance Inventory-Short Revised (WAI-SR)**, [52], a parent completed 12-item questionnaire assessing quality of therapeutic relationship consisting of three subscales (i) Goals measures agreement on intervention goals and outcomes, (ii) Tasks measures agreement on behaviours and thoughts underpinning the intervention process, and (iii) Bond measures mutual trust, acceptance and confidence.
- Structured worksheets recording participant intervention uptake, session attendance, retention, reasons for missed sessions and dropout.

Sample size

The primary *a priori* feasibility criterion was post-intervention retention rate of at least 65% [40,41]. A confidence interval approach was used to calculate a planned sample size of N=70 based on this primary criterion.[53] Using a 95% CI for the proportion of parents who completed treatment and an expected completion rate of 80% based on previous evaluations of HFP, it was determined that an HFP-M intervention sample size of n=35 would provide a sufficiently precise estimate (95% CI .67-.93). A sample size of n=70 was also sufficient to obtain stable estimates of population variances for future power calculations.[54]

For peer review only - http://bmjopen.bmj.com/site/about/guidelines.xhtml

Recruitment and consent procedures

Settings

Recruitment took place in two large UK NHS health services and concomitant local authority children's social care service in London (Site 1 and 2) located in areas of high mental health morbidity.

Identification and consent

Clinical keyworkers undertook exploratory discussion and provided written information to potentially eligible participants. With consent, contact details were provided to researchers, who provided information about study aims, eligibility criteria, procedures, and a Participant Information Sheet. One week later, parents were contacted to determine participation and obtain written informed consent.

Allocation and randomisation

Participants were allocated a unique, anonymised ID number and randomised to trial conditions between 11.05.16 and 29.03.17 by the Clinical Trials Unit, King's College, London. Allocation was communicated confidentially to the trial co-ordinator, other researchers remained blind to allocation status.

Data collection

Screening and assessments

Following consent, parents completed screening measures, and, when eligible, baseline measures in a standard sequence. Any parent discomfort was addressed sensitively and supportively. Subsequently, persistent and non-intrusive efforts were used to complete post-intervention and follow-up data collection. Participants were reimbursed £10 per hour for data completion at each time point.

Analysis

Statistical analysis was mainly descriptive using means and standard deviations for continuous data, or medians and range where data were skewed. Frequencies and proportions were used to describe categorical variables. Feasibility of trial retention was assessed using the proportion of a predetermined parameter and estimated 95% CIs. Clinical outcomes were analysed using ANCOVA models to estimate likely range of intervention effect, by assessing 95% CI, at post-treatment, with pre-randomization values as a covariate.[55] Follow-up data were not included in these analyses due to a smaller sample than planned. Standardised effect

For peer review only - http://bmjopen.bmj.com/site/about/guidelines.xhtml

sizes were calculated by dividing the estimated treatment effect by the standard deviation at baseline (Cohen's d). Population variances for future power calculations were determined using the upper 80th percentile of confidence intervals around the estimated population variance.[55].

Patient and public involvement

A service user organisation senior staff member was co-applicant who contributed to the research conception, funding and governance. A service user researcher was involved in the analysis, interpretation and dissemination of findings. A service user advisory panel advised on intervention development, recruitment and screening methods, outcome selection and interpretation of study findings.[56]

Independent ethical review and NHS research & development approval

Ethics approval was obtained from Health Research Authority South East Coast - Brighton & Sussex Research Ethics Committee (reference: 16/LO/0199). Research and development approvals were obtained from South London and Maudsley NHS Foundation Trust and Central and North-West London NHS Foundation Trust.

Serious adverse events

The chief investigator was responsible for reporting serious adverse events to the trial's independent Data Monitoring and Ethics Committee and responsible research ethics committee. No serious adverse events were reported.

Results

Feasibility evaluation

Participant recruitment took four months longer than planned due to delays in Site 2, resulting in a revised sample size of 48. Obtaining referring keyworkers data on the total number of service users they approached about trial participation proved impractical.

All referred service users (n=89, 100.0%) consented to research contact (see Figure 1). Adult mental health services (AMHS) referred 30 (33.7%) parents, child and adolescent mental health services (CAMHS) 29 (32.6%) parents, and children's social care (CSS) 30 (33.7%) parents. Sixty-five (73.0%) parents were referred by Site 1. Researchers made contact with 87 (97.7%) parents, requiring 1 to 13 separate communications per participant.

For peer review only - http://bmjopen.bmj.com/site/about/guidelines.xhtml

For peer review only

Sixty (69.7%) parents met initial criteria and completed screening. The most common reason for ineligibility was parents declining trial participation (n=12, 13.5%). Six parents were excluded due to child-related reasons, most commonly presence of child developmental disorder.

Forty-eight consenting participants met parent and child screening criteria, all of whom completed baseline measures, representing 80.0% of screened parents, 53.9% of referred parents and 68.8% of the planned sample. Five (8.3%) met neither parent nor child screening criteria, four (6.7%) did not meet SAPAS criterion, and three (5.0%) did not meet SDQ criterion.

Thirty-six (75.0%) trial participants were from Site 1, exceeding the site recruitment target. Site 2 recruitment (n=12) was 34.3% of that planned, due to delayed recruitment and lower service engagement. Eighteen (37.5%) participants were referred by AMHS, 16 (33.3%) CAMHS and 14 (29.2%) CSS.

There was a significant difference in trial condition uptake (HFP-M: n=21, 87.5%; Usual care n=15, 62.5%; $\chi 2=4.0$, df=1 (48), $p<0.05$). Modal duration between randomisation and starting HFP-M was 2 weeks (range 1-23 weeks). Parents declined HFP-M because one gave birth, another was in conflict with her partner about participation, and a third did not respond to persistent contact (see Figure 1). The modal duration before Usual care parenting session receipt was 5.5 weeks (range 1-17 weeks). The most common reason for declining the additional Usual care parenting session was that participants hoped to be allocated to HFP-M.

At post-intervention, 32 (66.7%, 95% C.I. 51.6% to 79.6%) participants completed post-intervention measures. The majority of participants who did not take up the intervention offered, mainly in the Usual care condition, did not complete post-intervention measures.

Twenty-one (65.6%) parents completing post-intervention measures were also assessed at follow-up, representing 43.8% of all trial participants. Post-intervention measures were completed a mean of 42.0 weeks (SD 14.6) after baseline, due to HFP-M delivery being lengthier in duration than planned. Follow-up measures were completed a mean of 20 weeks (SD 8.9) later. Researchers required one to eight participant contacts to arrange data completion post-intervention and at follow-up. Main reasons for non-completion included unable to contact participant, participant declining completion, and participant health and life circumstances.

For peer review only - http://bmjopen.bmj.com/site/about/guidelines.xhtml

There were less than 0.1% missing data items across measures at each time point.

Sample characteristics

Demographic characteristics

All 48 participants were the biological parent of the index child, one was a father (2.1%) (see Table 1). The majority were lone parents (n=31, 67.4%), mean age, 34.9 (SD. 7.1). The majority (n=28, 61.0%) were White British/Irish, with fewer Black/Black British (n=9, 19.6%) and Dual Heritage (n=6, 13.5%) parents. English was the most common first language (n=44, 91.7%). Twenty-five (55.6%) participants completed education at 18 years or younger. Four participants (8.9%) were higher education graduates.

Most participants (n=37, 80.4%) were not in paid employment. Nine participants (19.5%) had partners in paid employment, predominantly part-time (n=7, 15.2%). No parent was in paid employment in 30 (65.2%) households. Twenty-five (86.2%) parents had received a formal psychiatric diagnosis, the mean time since initial diagnosis was 9.7 (7.4) years.

Twenty-six (54.2%) index children were male, with a mean age of 7.8 (SD. 2.2) years. The median number of children in the home was 2, range 1-5 (see Table 1). Participants reported significant difficulties with anxiety/depression, pain/discomfort, and with a smaller proportion experiencing difficulties in undertaking everyday activities (see Table 1).

Clinical characteristics

Over eighty per cent of ECBI Problem and Intensity scores at baseline exceeded the clinical caseness cut-off, with similar caseness rates for CBCL-Int and PS (see Table 2). The most common baseline CAMC child-related parent concerns were conduct, self-regulation, parent-child relationships and emotional distress (see Table 3).

Trial interventions acceptability

HFP-M attendance

Of the 21 participants who accepted HFP-M, 13 (61.9%, 95% C.I. 38.4% to 81.9%) completed HFP-M within the trial period. Six (28.6%, 95% C.I. 11.3% to 52.2%) withdrew before completion due to acute adult mental health crisis and complex family circumstances unrelated to HFP-M receipt. Recruitment delay and longer than anticipated intervention duration resulted in two (9.5%, 95% C.I.: 1.2% to 30.4%) participants not completing HFP-M before trial conclusion. The mean number of HFP-M appointments offered was 15.8 (SD.

For peer review only - http://bmjopen.bmj.com/site/about/guidelines.xhtml

7.7) and mean number attended was 11.2 (SD. 6.3). HFP-M appointment attendance was 70.2%. Mean duration of HFP-M delivery was 28.4 (SD. 21.7) weeks.

HFP-M therapeutic alliance acceptability

Eighteen (85.3%) HFP-M participants completed post-intervention WAI-SR (mean total score=73.8, SD 10.4). Mean subscales scores were consistently in the upper end of the scale (Mean Tasks Subscale score, 24.9, SD. 3.5; Bond Subscale score, 24.9, SD. 3.9; Goals Subscale score.23.9, SD. 4.1).

Usual Care

All 15 participants accepting the additional parenting session completed. Due to lower retention, only six participants provided post-intervention WAI-SR data (mean total score= 56.2, SD 18.8). Mean subscales scores were consistently lower than the HFP-M condition (Mean Tasks Subscale score, 17.4, SD. 7.2; Bond Subscale score, 19.8, SD. 7.1; Goals Subscale score, 19.0, SD. 7.6).

There appeared to be a substantial difference in WAI-SR scores between the two conditions but there was insufficient Usual care data to test for a statistical difference.

No adverse events were reported over the course of the trial. Participant intervention withdrawal most frequently occurred due to deterioration in participant mental health and life circumstances, unrelated to trial participation.

Clinical outcomes

There were estimated mean improvements from baseline scores across a number of variables within both trial conditions (see Table 4). HFP-M mean differences exceeded those in Usual care on several outcomes, though these findings should be treated with caution given the wide confidence intervals.

Estimated effect sizes showed a general post-intervention advantage for HFP-M on a range of outcomes. Medium effects for child behavioural problem severity (ECBI Intensity, ES 0.4, CI, -0.3-1.1), and parenting satisfaction (KPSS, ES 0.4, CI, -0.3-1.1) were detected and a large effect size parent-reported reductions in concerns about their child (CAMC Problem 1, ES 1.2, CI, 0.4-2.0, CAMC Problem 2, ES 1.3, CI, 0.5-2.1). No effects were detected for parenting behaviour and adult mental health outcomes. Descriptive scrutiny of follow-up findings showed that outcome scores across both groups were generally maintained or continued to improve.

For peer review only - http://bmjopen.bmj.com/site/about/guidelines.xhtml

Estimates of standard deviation and upper confidence intervals (u80% CI) for future power calculations of main clinical outcomes are: ECBI Problem: SD 6.73 (u80%CI: 7.59), ECBI Intensity SD 33.60 ((u80%CI: 37.14), CAMC Problem 1: SD 15.25 (u80%CI: 17.44), and KPSS: 2.95 ((u80%CI: 3.33).

Conclusion

This study assessed feasibility, identified potential challenges and informed decision-making for the research and intervention methods of a definitive HFP-M trial.[28-30,39] Participant identification methods were successfully implemented across a wide range of mental health and CSS teams with over 90% of referred parents meeting dual child and parent screening criteria. Non-diagnostic eligibility criteria were acceptable and effective in recruiting an appropriate, multi-morbid sample.[57,58] Negative life events and disrupted family functioning, characteristic of the sample population commonly delayed screening, data collection and intervention delivery.[7] Nevertheless, participant retention exceeded the *a priori* primary feasibility criterion of 65%. Participants declining intervention conditions were less likely to be retained at follow-up. Participant recruitment was slower than planned, mainly due to operational difficulties in one site.

Participants' multi-morbid characteristics are commonly associated with poorer treatment engagement and outcomes.[59,60] Intervention uptake differed substantially between trial conditions. HFP-M was largely acceptable to participants but delivery was less efficient than planned, often due to session cancellation prompted by parents' life circumstances. The Usual care condition appeared to be less acceptable to participants, and potentially affected participant retention. Lower Usual care retention and acceptability rates may also reflect more widespread participant dissatisfaction associated with control condition allocation.[61,62]

Clinical effects, detected across trial conditions, with potential advantage for HFP-M, are welcome given the population's complex parenting impairments and negative treatment expectancies.[2,60] Effects were detected for child behaviour, parental child concerns and parenting satisfaction. Participant recruitment and retention affected final sample size, limited trial power and consequently affected interpretation of these findings. A definitive trial could potentially narrow child selection criteria to include only behaviour problems as parenting programmes have an established evidence base for this condition. Alternatively, the less substantial effects for child internalising difficulties may require strengthening HFP-M

For peer review only - http://bmjopen.bmj.com/site/about/guidelines.xhtml

content specifically in relation to these difficulties. Economic analysis also indicated potential cost advantages for HFP-M over usual care.[42]

HFP-M and Usual care conditions differed in duration and therapeutic intensity, which may account for potential outcome differences. Data collection relied on parent self-report, which is conventional given the poor reliability of child-report across the age group and costs associated with independent ratings.

A future definitive trial should be based on the assumption of a medium effect size for the primary outcome of child behaviour. Site engagement, resource allocation and keyworker training in participant identification and recruitment will be crucial to the recruitment of the larger sample required in a future trial. Embedded researchers to assist in caseload identification and undertake direct parent recruitment, not possible in this feasibility study, may promote trial recruitment flow. Participant retention, particularly parents allocated to a usual care condition, will continue to be challenging in a full trial. The population's complex personality difficulties and, typically, heightened sensitivity to rejection, underline the importance of researchers managing sensitively and effectively trial consent procedures, the emotional and practical consequences of random allocation as well as proactively maintaining communication and validating relationships with participants throughout trial duration, particularly for those allocated to usual care. Though not routinely available, the usual care condition could be augmented with an on-going parent support group to potentially increase equipoise, face validity and uptake, which may benefit trial retention.

HFP-M clinical and trial efficiency may be improved through more explicit, validating discussion with participants about the potential impact of life and personal circumstances on attendance, use of pre-emptive cancellation plans, and inclusion of inter-session contact using digital technology.[31,60,63]

Word count: 3993

For peer review only - http://bmjopen.bmj.com/site/about/guidelines.xhtml

References

[1] Fergusson, D., Horwood, L. & Ridder, E. (2005). Show me the child at seven: The consequences of conduct problems in childhood for psychosocial functioning in adulthood. *J Child Psychol Psyc,* 46, 837-*849.*

[2] Petfield, L., Startup, H., Drosher, H. & Cartwright-Hatton, S. (2015) Parenting in mothers with borderline personality disorder and impact on child outcomes. *Evid Based Ment Health*, 18(3), 67-75.

[3] Eyden, J., Winsper, C., Wolke, D., Broome, M. & MacCallum, F. (2016). A systematic review of the parenting and outcomes experienced by offspring of mothers with borderline personality pathology: Potential mechanisms and clinical implications. *Clin Psychol Rev*, 47, 85–105.

[4] Day, C., Harris., L., McCrone, P., McMurran, M., Moran, P., Morgan, L.et al. (2017) Feasibility trial of a psychoeducational intervention for parents with personality difficulties: The Helping Families Programme. *Contemporary Clinical Trials Communications*, 8, 67-74. doi.org/10.1016/j.conctc.2017.08.002.

[5] Ackerson, B. (2003). Coping with the dual demands of severe mental illness and parenting: The parents' perspective. *Fam Soc*, 84, 109-119.

[6] Newton-Howes, G., Weaver, T. & Tyrer, P. (2008). Attitudes of staff towards patients with personality disorder in community mental health teams. *Aust Nz J P Psychiat*, *42,* 572-577.

[7] Coid, J., Yang, M., Tyrer, P. & Roberts, A. (2006). Prevalence and correlates of personality disorder in Great Britain. *Brit J Psychiat*, 188, 423-431.

[8] Newton-Howes, G., Tyrer, P., Anagnostakis, K., Cooper, S., Bowden-Jones, O. & Weaver, T. (2010). The prevalence of personality disorder, its comorbidity with mental state disorders, and its clinical significance in community mental health teams. *Soc Psych Psych Epid, 45,* 453-460.

[9] McCrone, P., Patel, A., Knapp, M. & Lawton-Smith, S. (2008). *Paying the price: The cost of mental health care in England to 2026*. London: King's Fund. Available at: http://www.kingsfund.org.uk/sites/files/kf/Paying-the-Price-the-cost-of-mental-health-care-

For peer review only - http://bmjopen.bmj.com/site/about/guidelines.xhtml

England-2026-McCrone-Dhanasiri-Patel-Knapp-Lawton-Smith-Kings-Fund-May-2008_0.pdf

[10] Johnson, J., Cohen, P., Kasen, S., Ehrensaft, M. & Crawford, T. (2006). Associations of parental personality disorders and axis I disorders with childrearing behavior. *J Am Acad Child Psy*, 69, 336-350.

[11] Newman, L., Stevenson, C., Bergman, L. & Boyce, P. (2007). Borderline personality disorder, mother-infant interaction and parenting perceptions: Preliminary findings. *Aust Nz J Psychiat*, 41*,* 598-605.

[12] Green, H., McGinnity, A., Meltzer, H., Ford, T. & Goodman, R. (2005). *Mental health of children and young people in Great Britain, 2004.* Basingstoke: Palgrave Macmillan. Available at: http://www.esds.ac.uk/doc/5269/mrdoc/pdf/5269technicalreport.pdf

[13] Davis, H., Day, C., Cox, A. & Cutler L. (2000). Child and adolescent mental health needs assessment and service implications in an inner-city area. *Clin Child Psychol P,* 5, 169-*188.*

[14] Romeo R, Knapp M, Scott S. (2006). Economic cost of severe antisocial behaviour in childrenand who pays it. *Br J Psychiatry*, 188(6):547–553.

[15] Parsonage M, Khan L, Saunders (2014). *Building a better future: The lifetime costs of childhood behavioural problems and the benefits of early intervention*. Lond Cent

Ment Health.

[16] Department of Health, Department for Education (2017). *Transforming Children and Young People's Mental Health Provision: a Green Paper*. London: DoH/DfE.

[17] Social Care Institute for Excellence (2011) Think child, think parent, think family: a guide to parental mental health and child welfare. https://www.scie.org.uk/publications/guides/guide30/summary.asp. Last accessed 31.05.18.

[18] Allen, G. (2011). *Early intervention: The next steps.* London: Cabinet Office. Available at: http://www.dwp.gov.uk/docs/early-intervention-next-steps.pdf. Last accessed 22.11.18

[19] Mental Health Taskforce (2016). The Five Year Forward View of Mental Health. Mental Health taskforce. Available at: www.england.nhs.uk/mentalhealth/taskforce. Last accessed 22.11.18

For peer review only - http://bmjopen.bmj.com/site/about/guidelines.xhtml

[20] Parker, G., Beresford, B., Clarke, S. et al (2008). *Technical report for SCIE Research Review on the prevalence and incidence of parental mental health problems and the detection, screening and reporting of parental mental health problems*. Working Paper No. SCIE 2247: University of York.

[21] Wilson, R., Weaver, T., Michelson, D. & Day, C. (2018) Experiences of parenting and clinical intervention for mothers affected by personality disorder: a pilot qualitative study combining parent and clinician perspectives. *BMC Psychiatry,* 18,152. doi.org/10.1186/s12888-018-1733-8.

[22] National Institute for Health and Clinical Excellence (2013). *Antisocial behaviour and conduct disorders in children and young people: Recognition, intervention and management.* London: NICE.

[23] Michelson, D., Day, C., Dretzke., Davenport, C. & Barlow, J. (2013) Do evidence-based interventions work when tested in the "real world?" A systematic review and meta-analysis of parent management training for the treatment of child disruptive behavior. *Clin Child Fam Psych,* 16(1), 18-34.

[24] Reyno, S. & McGrath, P. (2006). Predictors of parent training efficacy for child externalizing behavior problems: A meta-analytic review. *J Child Psychol Psyc*, 47, 99-111.

[25] Lundahl, B., Risser, H. J., & Lovejoy, M. C. (2006). A meta-analysis of parent training: Moderators and follow-up effects. *Clin Psychol Rev*, *26*(1), 86-104.

[26] Dawe, S.*, &* Harnett, P. H. (2007). Improving family functioning in methadone maintained families: Results from a randomised controlled trial. *J Subst Abuse Treat***, 32,** 381-390.

[27] Thanhäuser M, Lemmer G, de Girolamo G, Christiansen H. (2017) Do preventive interventions for children of mentally ill parents work? Results of a systematic review and metaanalysis. *Curr Opin Psychiatry*;30(4):283–299.

[28] Craig, P., Dieppe, P., Macintyre, S., Michie, S., Nazareth, I. & Petticrew, M. (2008). *Developing and evaluating complex interventions: New guidance.* London: Medical Research Council.

For peer review only - http://bmjopen.bmj.com/site/about/guidelines.xhtml

[29] Hoffman, T., Glasziou, P., Boutron,, I., Milne, R., & Perera, R. (2014). Better reporting of interventions: Template for intervention description and replication (TIDieR) checklist and guide. *Brit Med J*, 348:g1687

[30] Eldridge, S., Chan, C., Campbell, M., Bond, C., Hopewell, S., Thabane, L et al. (2016). CONSORT 2010 statement: extension to randomised pilot and feasibility trial *Pilot Feasibility Stud*, 2:64.

[31] Bateman, A. W., & Fonagy, P. (2000). Effectiveness of psychotherapeutic treatment of personality disorder. *Brit J Psychiat*, 177(2), 138-143.

[32] McMurran, M., Crawford, M. J., Reilly, J.G., McCrone, P., Moran, P., Williams, H., et al. (2011). Psycho-education with problem solving (PEPS) therapy for adults with personality disorder: A pragmatic multi-site community-based randomised clinical trial. *Trials*, 12, 198.

[33] Day, C., Kowalenko, S., Ellis, M., Dawe, S., Harnett, P. & Scott, S. (2011). The Helping Families Programme: A new parenting intervention for children with severe and persistent conduct problems. *Child Adol Ment H-UK*, 16, 167-171.

[34] Day, C., Ellis., M. & Harris. (2015). *Family Partnership Model Reflective Practice Handbook*. 2nd Edn. South London & Maudsley NHS Foundation Trust/King's College, London.

[35] McMurran, M., Huband, N. & Overton, E. (2010). Non-completion of personality disorder treatments: A systematic review of correlates, consequences, and interventions. *Clin Psychol Rev*, 30, 277-287

[36] Tetley, A., Jinks, M., Huband, N., Howells., K & McMurran, M. (2012). Barriers to and facilitators of treatment engagement for clients with personality disorder: A Delphi survey. *Personal Ment Health*, 6, 97-110.

[37] Kazdin AE. Acceptability of alternative treatments for deviant child behavior. *J Appl Behav Anal*. 1980;13(2):259–273.

[38] Foster SL, Mash EJ. Assessing social validity in clinical treatment research: Issues and procedures. *J Consult Clin Psychol*. 1999;67(3):308.

[39] Bugge, C., Williams, B., Hagen, S., Logan, J., Glazener, C., Pringle, S., et al. (2013). A process for Decision-making after Pilot and feasibility Trials (ADePT): development

For peer review only - http://bmjopen.bmj.com/site/about/guidelines.xhtml

following a feasibility study of a complex intervention for pelvic organ prolapse. *Trials*, 14:353. doi: 10.1186/1745-6215-14-353. PubMed.

[40] Oud M, Arntz A, Hermens ML, Verhoef R, Kendall T. Specialized psychotherapies for adults with borderline personality disorder: A systematic review and meta-analysis. *Aust N Z J Psychiatry*. 2018;52(10):949–961.

[41] McMurran M, Huband N, Overton E. Noncompletion of personality disorder treatments: A systematic review of correlates, consequences, and interventions. *Clin Psychol Rev*. 2010;30(3):277–287.

[42] Day C, Briskman J, Crawford MJ, Foote L, Harris L, Boadu J, et al. Helping Families Programme-Modified: Development of a Specialised Parenting Intervention for Parents Affected by Severe Personality Difficulties and Randomised Feasibility Trial. Health Technol Assess. *Accepted for publication*.

[43] Moran, P., Leese, M., Lee, T., Walters, P., Thornicroft, G. & Mann, A. (2003). The Standardised Assessment of Personality - Abbreviated Scale (SAPAS): Preliminary validation of a brief screen for personality disorder. *Brit J Psychiat*, 183, 228-232.

[44] Goodman, R., Ford, T., Richards, H., Gatward, R. & Meltzer, H. (2000). The Development and Well-Being Assessment: Description and initial validation of an integrated assessment of child and adolescent psychopathology. *J Child Psychol Psyc*, 41, 645-655.

[45] Day, C., Michelson, M., Thomson, S., Penney, C. & Draper, L. (2012). Evaluation of a peer-led parenting intervention for child behaviour problems: A community-based randomised controlled trial. *Brit Med J*, 344, e1107.

[46] van Reenen, M. & Janssen, B. (2015). *EQ-5D-5L User Guide*. Available at: https://euroqol.org/wp-content/uploads/2016/09/EQ-5D-5L_UserGuide_2015.pdf [Accessed 29 May 2018].

[47] Eyberg, S. & Ross, A.W. (1978). Assessment of child behaviour problems: The validation of a new inventory. *J Clin Child Psychol*,7:2,113-116,DOI:10.1080/15374417809532835

[48] Achenbach, T. & Rescorla, L. (2001). *Manual for the ASEBA School-Age Forms and Profiles*. Burlington, VT: University of Vermont, Research Center for Children, Youth & Families.

For peer review only - http://bmjopen.bmj.com/site/about/guidelines.xhtml

[49] Arnold, D., O'Leary, S., Wolff, L. & Acker, M. (1993). The Parenting Scale: A measure of dysfunctional parenting in discipline situations. *Psychol Assessment*, 5, 137-144.

[50] James, D., Schumm, W., Kennedy, C., Grigsby, C., Shectman, K. & Nichols, C. (1985). Characteristics of the Kansas parental satisfaction scale among two samples of married parents. *Psychol Rep*, 57, 163-169.

[51] Müller, J., Postert, C., Beyer, T., Furniss, T. & Achtergarde, S. (2010). Comparison of eleven short versions of the Symptom Checklist 90-Revised (SCL-90-R) for use in the assessment of general psychopathology. *Psychol Assessment*, 32, 246-254.

[52] Hatcher, R., & Gillaspy, J. (2006). Development and validation of a revised short version of the Working Alliance Inventory. *Psychother Res*, 16, 12-25.

[53] Hertzog, M. (2009). Considerations in determining sample size for pilot studies. Res Nurs Health, 31, 180-191.

[54] Browne, R. (1995). On the use of a pilot sample for sample size determination. *Stat Med, 14*, 1933-1940.

[55] Brown, H. & Prescott. R. (2007). *Applied Mixed Models in Medicine. Second Edition.* New York: John Wiley & Sons.

[56] INVOLVE (2010). *Payment for Involvement: A guide for making payments to members of the public actively involved in NHS, public health and social care research*. INVOLVE: Eastleigh.

[57] McMurran, M., Delport, J., Wood, K., Jenkins, S., Wall, M. & Day, F. (2012). Recruitment to personality disorder treatment trials. *MHRJ*, 17, 119-128.

[58] Stines, L. & Feeny, N. (2008) Unique ethical concerns in clinical trials comparing psychosocial and psychopharmalogical interventions. *Ethics Behav*, 18:2-3, 234-246, DOI: 10.1080/10508420802064333

[59] Cooper, A & Conklin, L (2015) Dropout from individual psychotherapy for major depression: A meta-analysis of randomized clinical trials. *Clin Psychol Rev*, 40 57–65

[60] Barnicot, K., Katsakou, C., Marougka, S. & Priebe, S. (2011) Treatment completion in psychotherapy for borderline personality disorder – a systematic review and meta-analysis. *Acta Psychiat Scand*, 123: 327–338

For peer review only

For peer review only - http://bmjopen.bmj.com/site/about/guidelines.xhtml

[61] Lindström, D., Sundberg-Petersson, I., Adami, J. & Tönnesen, H. (2010) Disappointment and drop-out rate after being allocated to control group in a smoking cessation trial. *Contemp Clin Trials*, 1, 22-6. doi: 10.1016/j.cct.2009.09.003.

[62] Cook TD, Campbell DT: Quasi-experimentation: Design and Analysis Issues for Field Settings. Chicago, Rand McNally, 1979

[63] McMain, S., Boritz, T., & Leybman, M. (2015). Common strategies for cultivating a positive therapy relationship in the treatment of borderline personality disorder. *J Psychother Integr*, 25(1), 20-29.

For peer review only

For peer review only - http://bmjopen.bmj.com/site/about/guidelines.xhtml

Table 1: Participant demographic characteristics

Demographic characteristics		Baseline (Time 1)		
		Total	Intervention	Usual Care
Parent gender (Female) (n, %)		47 (97.9)	24 (100.0)	23 (95.8)
Parent age (yrs.)(mean, SD)		34.9 (7.1)	34.7 (7.5)	35.0 (6.9)
Received psychiatric diagnosis (n, %)		25 (86.2)	16 (88.9)	9 (81.8)
Psychiatric diagnosis duration (years, mean, SD)		9.7 (7.4)	8.6 (7.0)	10.9 (8.1)
Parent relationship to index child (biological parent) (n, %)		46 (100.0)	24 (100.0)	22 (100.0)
Index child gender (male) (n, %)		26 (55.3)	12 (50.0)	14 (60.9)
Index child age (mean, SD)		7.8 (2.2)	7.7 (2.0)	7.9 (2.2)
Number of children at home (median, range)		2 (1-5)	2 (1-5)	2 (1-5)
Not in paid employment		37 (80.4)	19 (79.2)	18 (81.8)
Lone parent (n, %)		31 (67.4)	14 (58.3)	17 (77.3)
Partner in employment		9 (19.5)	6 (25.0)	3 (12.5)
Parent education	Graduate	4 (8.9)	2 (8.3)	2 (9.5)
	University not completed	3 (6.7)	3 (12.5)	0 (0.0)
	Other e.g. NVQ	13 (28.9)	6 (25.0)	7 (33.3)
	Left school 18yrs	9 (20.0)	4 (16.7)	5 (23.8)
	Left school 16 years	7 (15.6)	4 (16.7)	3 (14.3)
	Left school under 16	9 (20.0)	5 (20.9)	4 (19.0)
Ethnicity	White UK/Irish	28 (61.0)	13 (54.2)	15 (68.2)
	Black UK/African Caribbean	9 (19.6)	7 (29.2)	2 (9.1)
	Dual heritage	6 (13.0)	3 (12.5)	3 (13.6)
	Black UK/African	2 (4.3)	0 (0.0)	2 (9.1)
	Other	1 (2.2)	1 (4.2)	0 (0.0)
Parent health status[1]	Mobility problems	9 (25.0)	4 (23.5)	5 (26.4)
	Problems in self-care washing & dressing	3 (8.3)	1 (5.9)	2 (10.5)
	Difficulties in undertaking usual activities	13 (36.1)	7 (41.1)	6 (31.6)
	Suffered pain/discomfort	17 (47.2)	6 (35.3)	11 (57.9)

[1] EQ-5D-5L health moderate/severe status

For peer review only - http://bmjopen.bmj.com/site/about/guidelines.xhtml

Table 2: Participant baseline clinical caseness

Measure	Baseline		
	Total	Intervention	Usual care
ECBI Problem Caseness (≥ 15) (n, %)	41 (89.1)	21 (87.5)	20 (90.9)
ECBI Intensity Caseness (≥ 131) (n, %)	39 (83.0)	19 (79.2)	20 (83.3)
CBCL-Int (t-score) Caseness (≥ 60) (n, %)	45 (95.8)	23 (95.8)	22 (95.7)
PS Caseness (≥ 3.2) (n, %)	35 (74.5)	18 (75.0)	17 (73.9)

Table 3: Parent-reported concerns about index child

Concern Category	Primary	Secondary	Tertiary	Total
Conduct problems[1]	18 (38.3)	27 (58.7)	24 (53.3)	69 (50.0)
Parent-child relationship & communication	16 (34.0)	2 (4.4)	4 (8.9)	22 (15.9)
Child self regulation[2]	4 (8.5)	11 (23.9)	7 (15.6)	22 (15.9)
Emotional distress[3]	8 (17.0)	4 (8.8)	7 (15.6)	19 (13.8)
Other[4]	1 (2.1)	2 (4.4)	1 (2.2)	4 (2.9)
School	0 (0.0)	0 (0.0)	2 (4.4)	2 (1.5)
Total	47	46	45	138

[1] including anger, tantrums, defiance, non-compliance, aggression, running away & lying; [2]including overactivity, poor concentration, overeating, & wetting; [3]including low mood, anxiety, low self-esteem; [4]including risk behaviours

For peer review only

For peer review only - http://bmjopen.bmj.com/site/about/guidelines.xhtml

Measure		Group	Baseline Mean (SD) (n)	Post-intervention Mean (SD) (n)	Follow-up Mean (SD) (n)	Baseline/Post-intervention Estimated mean difference (CI)	p	Effect Size
CAMC	Problem 1	Intervention	84.6 (16.0) (n=24)	45.2 (27.2) (n=18)	58.2 (29.5) (n=13)	18.633 (-40.177 to 2.910)	.087	1.2 (0.4-2.0)
		Usual Care	85.9 (14.7) (n=22)	63.1 (31.1) (n=14)	53.3 (34.4) (n=8)			
	Problem 2	Intervention	85.0 (19.1) (n=24)	51.1 (31.9) (n=18)	56.4 (35.5) (n=13)	22.486 (-44.318 to -.654)	.044	1.3 (0.5-2.1)
		Usual Care	82.9 (15.7) (n=22)	72.5 (26.0) (n=14)	66.5 (35.5) (n=8)			
	Problem 3	Intervention	81.0 (17.0) (n=24)	54.4 (33.8) (n=17)	42.8 (35.4) (n=13)	2.909 (-28.349 to 22.530)	.816	0.0 (-0.7-0.7)
		Usual Care	78.9 (18.5) (n=22)	57.3 (34.1) (n=14)	39.6 (24.3) (n=8)			
ECBI	Problem Score	Intervention	22.1 (7.4) (n=24)	17.9 (8.5) (n=15)	15.3 (9.4) (n=13)	1.559 (-4.24=55 to 7.374)	.585	0.1 (-0.7-0.88)
		Usual Care	22.43 (6.2) (n=21)	18.0 (11.5) (n=14)	18.8 (12.3) (n=8)			

For peer review only

For peer review only - http://bmjopen.bmj.com/site/about/guidelines.xhtml

	Intensity Score	Intervention	168.2 (35.9) (n=24)	142.4 (39.3) (n=24)	131.7 (43.0) (n=18)	12.866 (-4.24=55 to 7.374)	.233	0.4 (-0.3-1.1)
		Usual Care	169.5 (31.8) (n=23)	155.7 (49.6) (n=14)	148.9 (58.4) (n=8)			
CBCL-Int (t-score)		Intervention	72.9 (9.7) (n=24)	69.6 (10.4) (n=18)	68.2 (8.3) (n=13)	1.853 (-9.023 to 5.317)	.601	0.2 (-0.5-0.9)
		Usual Care	70.9 (11.9) (n=23)	70.9 (9.3) (n=14)	69.6 (8.6) (n=8)			
KPSS		Intervention	10.1 (3.1) (n=24)	12.9 (3.5) (n=18)	14.9 (3.8) (n=13)	1.177 (-1.260 to 3.615)	.331	0.4 (-0.3-1.1)
		Usual Care	10.9 (2.8) (n=23)	12.4 (3.9) (n=14)	13.6 (3.9) (n=7)			
PS		Intervention	111.0 (19.6) (n=24)	108.7 (16.5) (n=18)	98.3 (27.2) (n=13)	.210 (-14.776 to -15.196)	.977	0.0 (-0.7-0.7)
		Usual Care	113.5 (24.8) (n=23)	108.7 (28.9) (n=14)	98.1 (26.9) (n=7)			
SCL-27 Global Severity Index		Intervention	1.8 (1.1) (n=24	1.6 (0.8) (n=18)	1.9 (0.8) (n=13)	-.105 (-.599 to .389)	.666	-0.1 (-0.8-0.6)
		Usual Care	1.7 (0.8) (n=22)	1.7 (0.9) (n=14)	1.3 (1.0) (n=8)			

Table 4: Parent reported child, parenting and parent clinical outcomes (intention to treat analysis)

For peer review only - http://bmjopen.bmj.com/site/about/guidelines.xhtml

For peer review only

For peer review only - http://bmjopen.bmj.com/site/about/guidelines.xhtml

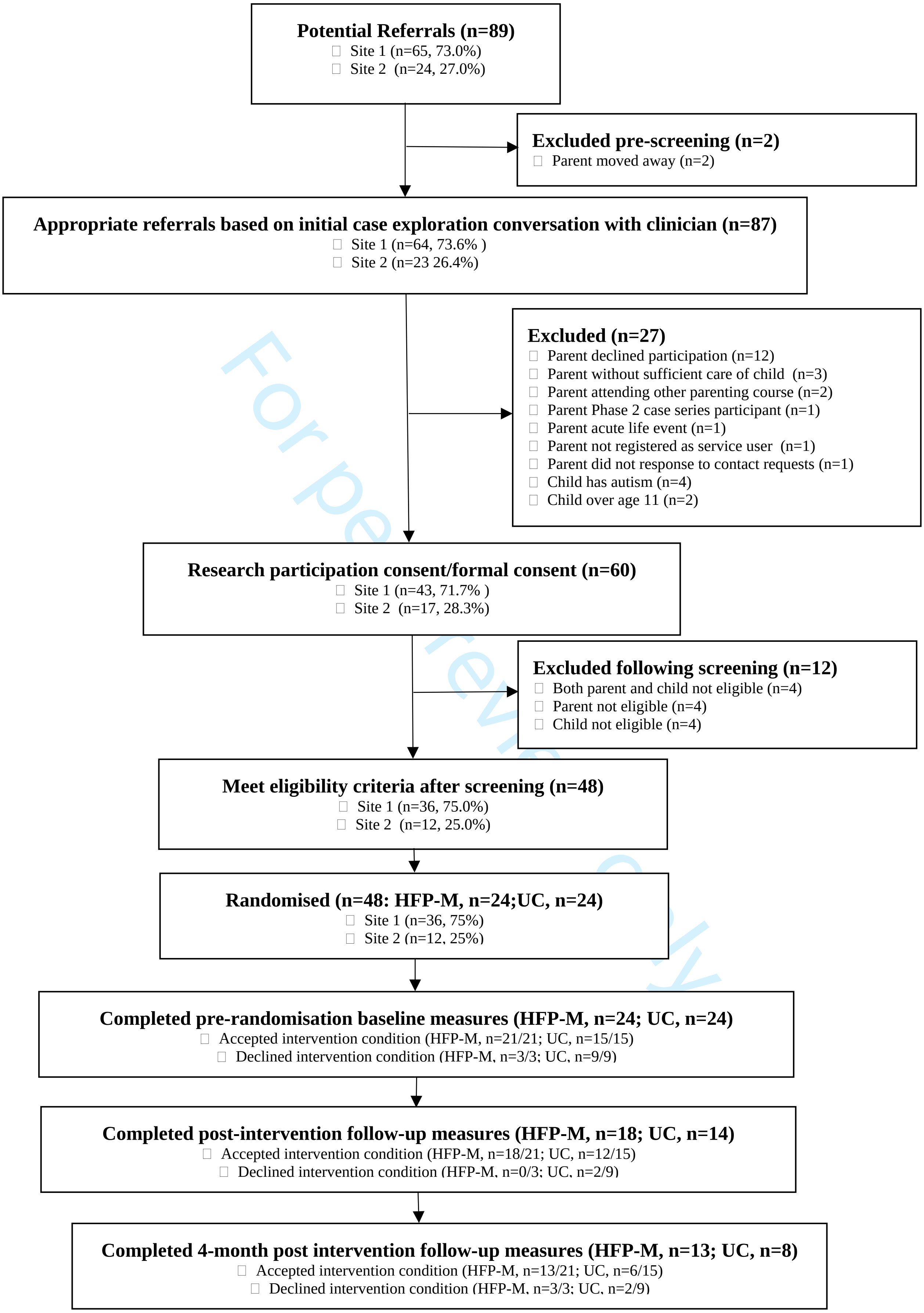

Figure 1: CONSORT diagram randomised feasibility trial recruitment and retention

For peer review only - http://bmjopen.bmj.com/site/about/guidelines.xhtml

For peer review only

For peer review only - http://bmjopen.bmj.com/site/about/guidelines.xhtml

CONSORT 2010 checklist of information to include when reporting a pilot or feasibility trial*

Section/Topic	Item No	Checklist item	Reported on page No
**Title and abstract**			
	1a	Identification as a pilot or feasibility randomised trial in the title	1
	1b	Structured summary of pilot trial design, methods, results, and conclusions (for specific guidance see CONSORT abstract extension for pilot trials)	2-3
**Introduction**			
Background and objectives	2a	Scientific background and explanation of rationale for future definitive trial, and reasons for randomised pilot trial	8-10
	2b	Specific objectives or research questions for pilot trial	10
**Methods**			
Trial design	3a	Description of pilot trial design (such as parallel, factorial) including allocation ratio	10
	3b	Important changes to methods after pilot trial commencement (such as eligibility criteria), with reasons	14, 15
Participants	4a	Eligibility criteria for participants	11
	4b	Settings and locations where the data were collected	13,14
	4c	How participants were identified and consented	13,14
Interventions	5	The interventions for each group with sufficient details to allow replication, including how and when they were actually administered	11,12
Outcomes	6a	Completely defined prespecified assessments or measurements to address each pilot trial objective specified in 2b, including how and when they were assessed	12,13
	6b	Any changes to pilot trial assessments or measurements after the pilot trial commenced, with reasons	N/A
	6c	If applicable, prespecified criteria used to judge whether, or how, to proceed with future definitive trial	10
Sample size	7a	Rationale for numbers in the pilot trial	13
	7b	When applicable, explanation of any interim analyses and stopping guidelines	N/A
Randomisation:			
Sequence generation	8a	Method used to generate the random allocation sequence	14
	8b	Type of randomisation(s); details of any restriction (such as blocking and block size)	10
Allocation concealment mechanism	9	Mechanism used to implement the random allocation sequence (such as sequentially numbered containers), describing any steps taken to conceal the sequence until interventions were assigned	14

For peer review only

Implementation	10	Who generated the random allocation sequence, who enrolled participants, and who assigned participants to interventions	14
Blinding	11a	If done, who was blinded after assignment to interventions (for example, participants, care providers, those assessing outcomes) and how	14
	11b	If relevant, description of the similarity of interventions	11,12
Statistical methods	12	Methods used to address each pilot trial objective whether qualitative or quantitative	14
**Results**			
Participant flow (a diagram is strongly recommended)	13a	For each group, the numbers of participants who were approached and/or assessed for eligibility, randomly assigned, received intended treatment, and were assessed for each objective	15,16,28
	13b	For each group, losses and exclusions after randomisation, together with reasons	15,16,28
Recruitment	14a	Dates defining the periods of recruitment and follow-up	14,15,16
	14b	Why the pilot trial ended or was stopped	N/A
Baseline data	15	A table showing baseline demographic and clinical characteristics for each group	29,30
Numbers analysed	16	For each objective, number of participants (denominator) included in each analysis. If relevant, these numbers should be by randomised group	15,16,28
Outcomes and estimation	17	For each objective, results including expressions of uncertainty (such as 95% confidence interval) for any estimates. If relevant, these results should be by randomised group	15,16,17,18, 19,31,32
Ancillary analyses	18	Results of any other analyses performed that could be used to inform the future definitive trial	N/A
Harms	19	All important harms or unintended effects in each group (for specific guidance see CONSORT for harms)	18
	19a	If relevant, other important unintended consequences	18
**Discussion**			
Limitations	20	Pilot trial limitations, addressing sources of potential bias and remaining uncertainty about feasibility	19,20
Generalisability	21	Generalisability (applicability) of pilot trial methods and findings to future definitive trial and other studies	20
Interpretation	22	Interpretation consistent with pilot trial objectives and findings, balancing potential benefits and harms, and considering other relevant evidence	19,20
	22a	Implications for progression from pilot to future definitive trial, including any proposed amendments	19,20
**Other information**			
Registration	23	Registration number for pilot trial and name of trial registry	ISRCTN14573230
Protocol	24	Where the pilot trial protocol can be accessed, if available	From Chief Investigator: rispin.1.day@kcl.ac.uk

For peer review only

Funding	25	Sources of funding and other support (such as supply of drugs), role of funders	National Institute of Health Research, Health Technology Assessment, Project Reference Number: 12/194/01
	26	Ethical approval or approval by research review committee, confirmed with reference number	Health Research Authority South East Coast - Brighton & Sussex Research Ethics Committee (reference: 16/LO/0199)

Citation: Eldridge SM, Chan CL, Campbell MJ, Bond CM, Hopewell S, Thabane L, et al. CONSORT 2010 statement: extension to randomised pilot and feasibility trials. BMJ. 2016;355.

*We strongly recommend reading this statement in conjunction with the CONSORT 2010, extension to randomised pilot and feasibility trials, Explanation and Elaboration for important clarifications on all the items. If relevant, we also recommend reading CONSORT extensions for cluster randomised trials, non-inferiority and equivalence trials, non-pharmacological treatments, herbal interventions, and pragmatic trials. Additional extensions are forthcoming: for those and for up to date references relevant to this checklist, see www.consort-statement.org.

For peer review only

BMJ Open

Randomised Feasibility Trial of the Helping Families Programme-Modified: An Intensive Parenting Intervention for Parents Affected by Severe Personality Difficulties

Journal:	*BMJ Open*
Manuscript ID	bmjopen-2019-033637.R1
Article Type:	Original research
Date Submitted by the Author:	18-Dec-2019

Complete List of Authors:	Day, Crispin; King's College London, ; South London and Maudsley NHS Foundation Trust,<br>Briskman, Jackie; King's College London<br>Crawford, Mike; Imperial College London, Centre for Psychiatry<br>Foote, Lisa; McPin Foundation<br>Harris, Lucy; South London and Maudsley NHS Foundation Trust,<br>Boadu, Janet; King's College London<br>McCrone, Paul; King's College London,<br>McMurran, Mary; University of Nottingham<br>Michelson, Daniel ; University of Sussex, School of Psychology<br>Moran, Paul; Univiersity of Bristol, Centre of Academic Mental Health, School of Social and Community Medicine; University of Bristol<br>Mosse, Liberty; King's College London<br>Scott, Stephen; King's College London<br>Stahl, Daniel; Department of Biostatistics Institute of Psychiatry, Psychology & Neuroscience (IoPPN), King's College London<br>Ramchandani, Paul; University of Cambridge<br>Weaver, Tim ; Middlesex University

**Primary Subject Heading**:	Mental health
Secondary Subject Heading:	Evidence based practice
Keywords:	Personality disorders < PSYCHIATRY, Parenting, Child behaviour, Child & adolescent psychiatry < PSYCHIATRY

SCHOLARONE™
Manuscripts

I, the Submitting Author has the right to grant and does grant on behalf of all authors of the Work (as defined in the below author licence), an exclusive licence and/or a non-exclusive licence for contributions from authors who are: i) UK Crown employees; ii) where BMJ has agreed a CC-BY licence shall apply, and/or iii) in accordance with the terms applicable for US Federal Government officers or employees acting as part of their official duties; on a worldwide, perpetual, irrevocable, royalty-free basis to BMJ Publishing Group Ltd ("BMJ") its licensees and where the relevant Journal is co-owned by BMJ to the co-owners of the Journal, to publish the Work in this journal and any other BMJ products and to exploit all rights, as set out in our licence.

The Submitting Author accepts and understands that any supply made under these terms is made by BMJ to the Submitting Author unless you are acting as an employee on behalf of your employer or a postgraduate student of an affiliated institution which is paying any applicable article publishing charge ("APC") for Open Access articles. Where the Submitting Author wishes to make the Work available on an Open Access basis (and intends to pay the relevant APC), the terms of reuse of such Open Access shall be governed by a Creative Commons licence – details of these licences and which Creative Commons licence will apply to this Work are set out in our licence referred to above.

Other than as permitted in any relevant BMJ Author's Self Archiving Policies, I confirm this Work has not been accepted for publication elsewhere, is not being considered for publication elsewhere and does not duplicate material already published. I confirm all authors consent to publication of this Work and authorise the granting of this licence.

For peer review only

Randomised Feasibility Trial of the Helping Families Programme-Modified: An Intensive Parenting Intervention for Parents Affected by Severe Personality Difficulties

Crispin Day*[a], Jackie Briskman[b], Mike J Crawford[c], Lisa Foote[d], Lucy Harris[a], Janet Boadu[e], Paul McCrone[e], Mary McMurran[g], Daniel Michelson[h], Paul Moran[i], Liberty Mosse[a], Stephen Scott[j], Daniel Stahl[k], Paul Ramchandani[l], & Timothy Weaver[m].

*Corresponding author

[a] CAMHS Research Unit, Centre for Parent and Child Support, South London and Maudsley NHS Foundation Trust, Michael Rutter Centre, De Crespigny Park, London SE5 8AF, UK. Email: crispin.1.day@kcl.ac.uk

[b] Dept. of Psychology, Institute of Psychiatry, Psychology and Neuroscience, King's College London, De Crespigny Park, London SE5 8AF, UK.

[c] The Centre for Psychiatry, Imperial College London, 7th Floor Commonwealth Building, Hammersmith Hospital Campus, London W12 0NN, UK.

[d] McPin Foundation, 32-36 Loman St, London SE1 0EH, UK.

[e] King's Health Economics, P024 David Goldberg Centre, Institute of Psychiatry, Psychology & Neuroscience, King's College London, De Crespigny Park, London SE5 8AF, UK.

[f] Institute of Lifecourse Development, University of Greenwich

[g] Institute of Mental Health, University of Nottingham, Triumph Road, Nottingham, NG7 2TU, UK.

[h] School of Psychology, University of Sussex, Falmer, Brighton BN1 9QH

[i] Centre for Academic Mental Health, Department of Population Health Sciences, Bristol Medical School, University of Bristol, Bristol BS8 2BN, UK.

[j] Department of Child and Adolescent Psychiatry, Institute of Psychiatry, Psychology and Neuroscience, King's College London, De Crespigny Park, London SE5 8AF, UK.

[k] Department of Biostatistics, Institute of Psychiatry, Psychology and Neuroscience, King's College London, De Crespigny Park, London SE5 8AF, UK.

[l] PEDAL Research Centre, Faculty of Education, University of Cambridge, UK.

[m] Department of Mental Health, Social Work and Integrative Medicine, School of Health and Education, Middlesex University, The Burroughs, London NW4 4BT, UK.

For peer review only

For peer review only - http://bmjopen.bmj.com/site/about/guidelines.xhtml

Randomised Feasibility Trial of the Helping Families Programme-Modified: An Intensive Parenting Intervention for Parents Affected by Severe Personality Difficulties

Abstract

Background

Specialist parenting intervention could improve coexistent parenting and child mental health difficulties of parents affected by severe personality difficulties.

Objective

Conduct a feasibility trial of Helping Families Programme-Modified (HFP-M), a specialist parenting intervention.

Design

Pragmatic, mixed-methods trial, 1:1 random allocation, assessing feasibility, intervention acceptability and outcome estimates.

Settings

Two NHS health trusts and local authority children's social care.

Participants

Parents: (i) primary caregiver, (ii) 18-65 years, (iii) severe personality difficulties, (iv) proficient English, and (v) capacity for consent. Child: (i) 3-11 years, (ii) living with index parent, and (iii) significant emotional/behavioural difficulties.

Intervention

HFP-M: 16-session home-based intervention using parenting and therapeutic engagement strategies. Usual care: standard care augmented by single psychoeducational parenting session.

Outcomes

For peer review only

For peer review only - http://bmjopen.bmj.com/site/about/guidelines.xhtml

Primary feasibility outcome: participant retention rate. Secondary outcomes: (i) rates of recruitment, eligibility and data completion, and (ii) rates of intervention acceptance, completion and alliance (Working Alliance Inventory-Short Revised). Primary clinical outcome: child behaviour (Eyberg Child Behavior Inventory). Secondary outcomes: child mental health (Concerns About My Child, Child Behavior Checklist-Internalising Scale), parenting (Arnold-O'Leary Parenting Scale, Kansas Parental Satisfaction Scale), and parent mental health (Symptom Checklist-27). Quantitative data were collected blind to allocation.

Results

Findings broadly supported non-diagnostic selection criterion. Of 48 participants recruited, 32 completed post intervention measures at mean 42 weeks later. Participant retention exceeded *a priori* rate (HFP-M=18; Usual care=14; 66.7%, 95% CI 51.6%-79.6%). HFP-M was acceptable, with delivery longer than planned. Usual care had lower alliance rating. Child and parenting outcome effects detected across trial arms with potential HFP-M advantage (ES range: 0.0-1.3).

Conclusion

HFP-M is an acceptable and potentially effective specialist parenting intervention. A definitive trial is feasible, subject to consideration of recruitment and retention methods, intervention efficiency and comparator condition. Caution is required in interpretation of results due to reduced sample size. No serious adverse events reported.

Trial registration

ISRCTN14573230

For peer review only

For peer review only - http://bmjopen.bmj.com/site/about/guidelines.xhtml

Article Summary

Strengths and Limitations of this Study

- This randomised trial assessed the feasibility of a specialist parenting intervention for coexistent mental health problems of parents affected by severe personality difficulties and their children.
- Findings provide useful evidence to support further evaluation of this specialist parenting intervention within a definitive trial, with modifications required to improve intervention efficiency, augmented usual care condition acceptability, and participant enrolment and retention.
- Caution is required in interpretation of results due to reduced sample size.
- The trial population's complex personality difficulties underline the importance of effective and sensitive management of trial consent procedures, random allocation and ongoing engagement of participants, particularly for those allocated to the usual care condition.

Keywords

Child behaviour, parenting, child & adolescent psychiatry, personality disorder

Funding

This work was supported by National Institute of Health Research, Health Technology Assessment, Project Reference Number: 12/194/01.

For peer review only

For peer review only - http://bmjopen.bmj.com/site/about/guidelines.xhtml

Contributions of the Authors

Dr Crispin Day, Head, Centre for Parent and Child Support, South London and Maudsley NHS Foundation Trust and Head, CAMHS Research Unit, King's College, London, was the chief investigator and responsible for the overall conception, design, data acquisition, analysis, interpretation of findings at each phase of this research. He is responsible for the overall content of this report.

Ms Jackie Briskman, Department of Psychology, King's College, London, was the senior research trial coordinator and was responsible for data acquisition, analysis, interpretation, drafting findings and revising this report.

Professor Mike J. Crawford, Professor in Mental Health Research, Centre for Psychiatry, Imperial College, London was a co-applicant and contributed to the overall conception, design and interpretation of findings at each phase of this research. He provided an expert contribution about personality disorder. He contributed to critically revising this report.

Ms Lisa Foote, McPin Foundation, was a service user researcher and representative in this research. She contributed to the design, data acquisition, analysis, interpretation and drafting of findings. She contributed to critically revising this report

Dr Lucy Harris, Deputy Head, Centre for Parent and Child Support, South London and Maudsley NHS Foundation Trust, was responsible for the development and revision of Helping Families Programme-Modified and supervised trial therapists. She contributed to critically revising this report.

Ms Janet Boadu is a researcher at King's Health Economics, King's College, London, co-conducted the health economic analysis and drafted findings for this report.

Professor Paul McCrone, Professor of Health Economics and Director of King's Health Economics, King's College, London, was a co-applicant and contributed to the overall conception, design and interpretation of findings at each phase of this research. He led the health economic component of the research and contributed to critically revising this report.

Professor Mary McMurran, Professor at the Institute of Mental Health, University of Nottingham was a co-applicant and contributed to the overall conception, design and interpretation of findings at each phase of this research. She provided an expert contribution

For peer review only - http://bmjopen.bmj.com/site/about/guidelines.xhtml

about personality disorder. She was a member of the Manualisation Working Group, contributed to research therapist training and contributed to critically revising this report.

Dr Daniel Michelson, Senior Lecturer in Clinical Psychology, School of Psychology, University of Sussex, was a co-applicant and worked closely with the chief investigator on the overall conception and design of this research. He was senior research coordinator during the intervention development phase leading up to this trial and contributed to critically revising this report.

Professor Paul Moran, Professor of Psychiatry, Population Health Sciences, University of Bristol, was a co-applicant and contributed to the overall conception, design and interpretation of findings at each phase of this research. He provided an expert contribution about personality disorder. He contributed to research therapist raining and critically revised this report.

Ms Liberty Mosse, CAMHS Research Unit, King's College, London, was a researcher worker on this study. She was responsible for Phase 3 data acquisition, analysis and interpretation of findings. She completed the majority of qualitative data acquisition and led its analysis under the direction of the chief investigator. She contributed to critically revising this report

Professor Stephen Scott, Professor of Child Health and Behaviour, King's College, London, was a co-applicant and contributed to the overall conception, design and interpretation of findings at each phase of this research. He provided an expert contribution about parenting and parenting programmes. He contributed to critically revising this report.

Dr Daniel Stahl, Reader in Biostatistics, Biostatistics and Health Informatics, King's College, London, was a co-applicant and contributed to the overall conception, design and interpretation of findings at each phase of this research. He provided an expert contribution to the design, analysis and interpretation of quantitative components of the research. He contributed to critically revising this report.

Professor Paul Ramchandani, LEGO Professor of Play in Education, Development and Learning, Faculty of Education, University of Cambridge, was a co-applicant and contributed to the overall conception, design and interpretation of findings at each phase of this research.

For peer review only - http://bmjopen.bmj.com/site/about/guidelines.xhtml
For peer review only

He provided an expert contribution about child mental health and co-ordinated the research in CNWL. He contributed to critically revising this report.

Dr Timothy Weaver, Associate Professor in Mental Health, Mental Health Social Work & Interprofessional Learning, Middlesex University, London, was a co-applicant and contributed to the overall conception, design and interpretation of findings at each phase of this research. He provided an expert contribution about qualitative design, data acquisition and interpretation. He contributed to critically revising this report.

Manual and Data Availability

All manuals can be obtained from the corresponding author. All data requests should be submitted to the corresponding author for consideration. Access to anonymised data may be granted following review.

Acknowledgements

We are indebted to the parents, practitioners, clinicians and service managers who worked with us on this trial, particularly Ruth Wilson. We would particularly like to thank Sarah Inkpen, Bethan Stevenson and Chelsea McCorry for their help with trial findings. Peter Fonagy and Philip Graham for overseeing the governance of the trial.

Competing Interests

Dr Crispin Day is the lead developer of two parenting programmes used in this report: Helping Families Programme and Empowering Parents Empowering Communities.

Prof Mike Crawford has previously received research grant funding on behalf of Imperial College London from the National Institute for Health Research.

Dr Lucy Harris is a co-developer of the Helping Families Programme.

Prof Mary McMurran was an author of the Psychoeducation plus Problems Solving (PEPS) intervention for adults with personality disorder. PEPS helped to inform the modified HFP.

Dr. Paul Moran reports personal fees from a talk given at 4th. Bergen International Conference on Forensic Psychiatry, 2016 Outside of the submitted work, Dr Moran led the development of the SAPAS, the personality disorder screen used in this study.

For peer review only - http://bmjopen.bmj.com/site/about/guidelines.xhtml

Introduction

Mental ill health is the largest cause of disability, with three-quarters of lifetime disorders starting during childhood.[1] Children of parents affected by severe personality difficulties are at particular risk due to the impact of parents' symptoms and associated impairment on parenting capacity.[2-4] Lack of evidence-based treatments, under-developed care pathways and stigma result in poorer immediate and longer-term child outcomes, increase intergenerational transmission of mental health difficulties and perpetuate social disadvantage.[5,6]

Severe personality difficulties, including personality disorders, affect over 4% of UK adults and 40% of mental health service users, at least one-quarter of whom are parents.[7,8] Per annum UK treatment costs exceed £70 million, with wider societal costs estimated at £8 billion per year.[9] Characterised by problematic interpersonal relationships, emotional dysregulation and poor impulse control, severe personality difficulties are associated with insensitive and intrusive interactions with offspring, family hostility, inconsistent and unpredictable family routines that undermine affectionate, stable, and responsive parenting required for healthy child development.[2-4] Affected parents are likely to suffer higher parenting stress and lower satisfaction, exacerbating underlying mental health difficulties.[10,11]

One in ten UK children suffer mental health disorders, with children of parents with severe personality difficulties at substantially higher risk of intergenerational transmission, most commonly behavioural disorders.[12,13] These childhood disorders are associated with academic failure, school exclusion, maltreatment, self-harm and gang affiliation, with increased life-time risk of comorbid mental and physical health conditions, drug misuse, offending and worklessness.[1,12,13] Annual UK public service costs for severe behavioural problems are estimated at £5,000 per child including £1,400 health costs. Lifetime estimated costs range from £85,000 (moderate case) to £260,000 (severe case).[14,15]

Concerted preventative and early intervention during pregnancy, infancy and childhood is warranted.[16] Effective care models for co-occurring child and parental mental health problems are significant health policy and research priorities.[17-19] Nevertheless, routine care is highly variable, generally focused on the needs of *either* the adult *or* the child,[17] resulting in under-identification, poor understanding of the interrelationship between child

For peer review only - http://bmjopen.bmj.com/site/about/guidelines.xhtml

and parent difficulties, and misattribution of parenting difficulties to adult mental health symptoms *per se*.[20] Affected parents can be reluctant to engage due to stigma, treatment scepticism and the interpersonal difficulties and adverse life circumstances associated with personality difficulties.[21]

Parenting and parent factors are central to much effective child mental health treatment and problem remediation. Parenting programmes are the recommended cost-effective treatment for child behaviour problems.[22,23] However, non-specialised parenting programmes, not specifically designed for parents affected by mental health difficulties, often result in poorer engagement, acceptability and outcomes.[24,25] Specialist interventions for some parental mental health conditions, such as depression, substance misuse and eating disorders, have demonstrated improved outcomes and reduced intergenerational transmission, by up to 40%.[26,27] Specialist programmes for parents affected by personality difficulties are at an earlier stage of evidence production.

Helping Families Programme-Modified (HFP-M) was developed as a specialist, intensive parenting intervention to address this need and service gap. It aims to improve immediate child and parenting outcomes with longer-term potential to reduce intergenerational transmission and psychosocial adversity within affected families.[4] Guided by recommended frameworks, HFP-M development synthesised two existing evidence-based interventions, incorporating relevant clinical practice recommendations and service user consultation.[22,28-33] Consistent with other promising programmes aiming to improve parenting and child outcomes in high risk groups, HFP-M is based on a transtheoretical model of parenting drawing on attachment, social learning and cognitive-affective theories and methods.[34,35] HFP-M does not target personality difficulties *per se* but aims to improve the ways that these characteristics affect parenting behaviour, emotional regulation, parent-child relationships and lead to adverse child outcomes.

HFP-M has three structured components [36]: (i) Core Therapeutic Process: including partnership and goal-based methods to promote collaborative relational engagement, shared formulation, empathic parent validation and crisis management;[36] (ii) Parent Groundwork: including emotion-focussed, cognitive, behavioural and interpersonal strategies to manage parental emotional dysregulation and hostility while relating to their children and undertaking parenting tasks, and (iii) Parenting Strategies: including consistent use of positive parenting

For peer review only - http://bmjopen.bmj.com/site/about/guidelines.xhtml

skills, such as, praise, consequences and limit setting, and relational and affective parenting methods such as emotionally responsive, warm care-giving and reflective function.

Definitive trials require successful recruitment, retention, and intervention acceptability.[30, 37-41] Participant retention underpins trial validity. Lower rates reduce power, undermine interpretation of findings and increase costs. HFP-M retention, and initial recruitment was expected to be affected by: (i) participants' core clinical features, (ii) greater exposure to family stress, negative life events, lower levels of social support and co-morbid mental health conditions, and (iii) under-identification of need within routine care, negative referrer expectancies, lower service engagement and attendance. These factors are reflected in evidence derived from 45 personality disorder treatments trials reported in two systematic reviews indicating a median participant non-completion rate of 35%.[42,43] Pre-feasibility trial case series findings, consultation with service user, clinicians and research ethics indicated that initial plans for trial recruitment based on personality disorder research diagnosis was unlikely to be viable for practical, participant acceptability and ethical reasons.[21]

To be useful and effective in practice, interventions need to demonstrate both clinical efficacy and user acceptability. Acceptability refers to service user judgements across four inter-related domains of intervention satisfaction: (i) relevance, (ii) content and procedures, (iii) clinician/provider characteristics and (iv) outcome suitability. [39,40]

This study reports quantitative findings from a randomised feasibility trial of HFP-M, based on a published protocol.[4] The trial aimed to assess research and clinical feasibility of HFP-M for a target population with co-existing parent personality difficulties and child mental health difficulties with findings being used to inform the design of a full-scale trial.

The primary feasibility outcome was a participant retention rate of at least 65% post-intervention. Secondary feasibility outcomes were rates of: (i) participant identification and recruitment, (ii) data collection, and (iii) intervention use, uptake and acceptability. Primary clinical outcome was child behaviour. Secondary clinical outcomes included parental child concerns, child internalising difficulties, parenting behaviour, satisfaction and psychological well-being. The trial sought to produce effect sizes and variance estimates for child and parent outcomes necessary to power a full-scale trial.

For peer review only - http://bmjopen.bmj.com/site/about/guidelines.xhtml

The findings of a parallel qualitative process evaluation investigating the influence of contextual factors on trial and intervention implementation and outcome generation are published elsewhere, as are the full findings of preliminary intervention costs and estimates of cost-effectiveness.[44]

Method

Design

Mixed-method, two-arm, parallel feasibility trial with random allocation in a 1:1 ratio to either: (i) HFP-M, or (ii) Usual care.

Quantitative data were collected at pre-randomisation baseline (Time 1), post-intervention (Time 2), six months from baseline, and follow-up (Time 3), 4-month, ten months from baseline.

Eligibility criteria

Parent: (i) primary parental caregiver for index child, (ii) aged 18-65, (iii) experiencing severe personality difficulties, assessed by self-administered Standardised Assessment of Personality-Abbreviated Scale (SAPAS) score of ≥3, the optimal cut-point for the intended sample population,[45] (iv) proficient written and spoken English, and (v) capacity to provide informed consent.

Child: (i) aged 3-11, (ii) living with index parent, and (iii) experiencing significant emotional/behavioural difficulties, assessed by Strengths and Difficulties Questionnaire Total Score of ≥17.[46]

Exclusion criteria: Parent (i) co-existing psychosis, (ii) engagement in another structured parenting intervention, (iii) inpatient status, or (iv) insufficient language/cognitive abilities. Child (i) pervasive developmental disorder, (ii) not residing with index parent, or (iii) considered for/subject to child protection supervision.

Interventions

HFP-M Intervention

16-session home-based 1:1 parenting intervention for parents with severe personality difficulties, including personality disorder. Session modules proceeded iteratively to (i) establish effective, validating collaborative partnership, (ii) develop shared understanding of

For peer review only - http://bmjopen.bmj.com/site/about/guidelines.xhtml

severe parental personality difficulties' impact on parenting, child functioning and family ecology, (iv) implement parent quick wins, parenting and child intervention goals, (v) use evidence-based parenting and parent self-care strategies to achieve agreed goals, and (vi) recurrent review of goals and therapeutic partnership. Six trial therapists received eight, three-hour, training sessions provided by HFP-M programme developers and clinical experts. Trial therapists completed structured checklists and received fortnightly supervision from experienced HFP-M clinicians to support clinical implementation and fidelity.

Usual care

No systematised parenting pathway was typically provided for the participant population. To provide consistent, low intensity support, participants could receive an additional home-based one-to-one parent information and support session. Derived from the evidence-based Empowering Parents Empowering Communities parenting programme, [47] session content included: (i) brief exploration of parenting and child needs, family support, parent priorities and goals, and (ii) focus on one parent priority topic, selected from Being Good Enough, Listening to My Child, Praising My Child, Taking Care of Myself, Understanding My Child's Behaviour, My Child's Emotion or Playing Together. The additional session was delivered by three trained parent practitioners, who received ongoing supervision to support implementation and fidelity.

Concomitant interventions

Both HFP-M and the single Usual care parent support session were provided in addition to existing medical, psychosocial and educational support and treatment services used by participating parents and their families. A joint-working protocol specified procedures for care co-ordination and information sharing between trial therapists and routine services.

Measures

Participant characteristics

Descriptive data were collected on parent and child age, gender and ethnicity, family household composition, participant diagnostic status, and family socio-economic status. Data from EQ-5D,[48,49] provided information about parent and child health and disability.

For peer review only

Feasibility evaluation

Structured record sheets, completed prospectively by research staff and trial therapists, documented: (i) participant identification and verbal consent, (ii) screening, eligibility, informed written consent, randomisation, and reasons for non-participation, and (iii) data collection and missing data.

Clinical outcomes

- **Eyberg Child Behavior Inventory (ECBI)**,[50] a 36-item questionnaire assessing intensity and number of disruptive behaviour problems in 2-16 year-olds, providing a comprehensive measure of child behaviour difficulties. Intensity Scale score of ≥131 indicates significant severity. Problem Scale score of ≥15 indicates significant number of problems.
- **Concerns About My Child (CAMC)**,[47] a visual analogue scale (0-100) rating three parental concerns about their child. Concerns nominated at baseline were re-rated at each time point, providing a sensitive, individualised index of change.
- **Child Behavior Checklist-Internalising Scale (CBCL-Int)**,[51] a 32-item questionnaire assessing internalising problems in 6-18 year-olds, with an alternate 36-item version for children aged 1½-5 years. Standardised T-scores combine results from both versions. A score of ≥60 indicates clinical caseness.
- **Arnold-O'Leary Parenting Scale (PS)**,[52] a 30-item questionnaire assessing dysfunctional parental discipline behaviour for children aged 2-16 years, which correlates with more time-consuming observational ratings. A score of ≥3.2 differentiates clinic and non-referred children.
- **Kansas Parental Satisfaction Scale (KPSS)**,[53] a 3-item scale providing a brief measure of parenting stress and satisfaction.
- **Symptom Checklist-27 (SCL-27)**,[54] a 27-item questionnaire assessing psychological symptoms in adults that provides a Global Severity Index (GSI) of psychopathology.

Intervention acceptability:

- **Working Alliance Inventory-Short Revised (WAI-SR)**,[55], a parent completed 12-item questionnaire assessing therapeutic relationship quality consisting of three subscales: (i) Goals, measuring agreement on intervention goals and outcomes, (ii)

For peer review only - http://bmjopen.bmj.com/site/about/guidelines.xhtml

Tasks, measuring agreement on behaviours and thoughts underpinning intervention process, and (iii) Bond, measuring mutual trust, acceptance and confidence.

- Structured worksheets recorded intervention uptake, attendance, retention, reasons for missed sessions and dropout.

Health economic:

- **Client Service Receipt Inventory (CSRI)**, [56] a schedule adapted to measure the use of services by caregivers and children.
- **EQ-5D-5L and EQ-5D-Y**,[48,49] a generic measure of health-related quality of life used to generate quality-adjusted life years. EQ-5D-Y is adapted for younger respondents.

Sample size

The primary feasibility criterion was post-intervention retention rate of at least 65% [42,43]. A confidence interval approach was used to calculate a planned sample size of N=70.[57] Using a 95% CI for the proportion of parents who completed treatment and an expected completion rate of 80% based on previous evaluations of HFP, it was determined that an HFP-M intervention sample size of n=35 would provide a sufficiently precise estimate (95% CI .67-.93). A sample size of n=70 was also sufficient to obtain stable estimates of population variances for future power calculations.[58]

Recruitment and consent procedures

Settings

Recruitment took place in two large UK NHS health services and concomitant local authority children's social care service in London (Site 1 and 2), located in areas of high mental health morbidity.

Identification and consent

Clinical keyworkers undertook exploratory discussion and provided written information to potential participants. With consent, contact details were provided to researchers, who provided information about study aims, eligibility criteria, procedures, and a Participant Information Sheet. One week later, parents were contacted to determine participation and obtain written informed consent.

For peer review only - http://bmjopen.bmj.com/site/about/guidelines.xhtml

Allocation and randomisation

Participants were allocated a unique, anonymised ID number and randomised to trial conditions between 11.05.16 and 29.03.17 by Clinical Trials Unit, King's College, London. Allocation was communicated confidentially to the trial co-ordinator, other researchers remained blind to allocation.

Data collection

Screening and assessments

Following consent, parents completed screening measures, and, when eligible, baseline measures in a standard sequence. Any parent discomfort was addressed sensitively and supportively. Persistent, non-intrusive efforts were used to complete post-intervention and follow-up data collection. Participants were reimbursed £10 per hour for data completion at each point.

Analysis

Statistical analysis was mainly descriptive using means and standard deviations for continuous data, or medians and range where data were skewed. Frequencies and proportions were used to describe categorical variables. Feasibility of trial retention was assessed using the proportion of a predetermined parameter and estimated 95% CIs. Clinical outcomes were analysed using ANCOVA models to estimate likely range of intervention effect, by assessing 95% CI, at post-treatment, with pre-randomization values as a covariate.[59] Follow-up data were not included in these analyses due to a smaller sample than planned. Standardised effect sizes were calculated using Cohen's d. Given the complexity of coexistent parent and child mental health difficulties, the smallest change in outcome identified as clinically important is equivalent to a small effect size. Population variances for future power calculations were determined using the upper 80th percentile of confidence intervals around the estimated population variance.[59].

Patient and public involvement

A senior staff member of a national service user organisation was a co-applicant and contributed to research conception, planning and governance. A service user researcher was involved in the analysis, interpretation and dissemination of findings. A service user panel

For peer review only - http://bmjopen.bmj.com/site/about/guidelines.xhtml

advised on trial planning, intervention methods, outcome selection and interpretation of findings.[60]

Independent ethical review and NHS research & development approval

Ethics approval was obtained from Health Research Authority South East Coast - Brighton & Sussex Research Ethics Committee (reference: 16/LO/0199). Research and development approvals were obtained from South London and Maudsley NHS Foundation Trust and Central and North-West London NHS Foundation Trust.

Serious adverse events

The chief investigator was responsible for reporting serious adverse events to the trial's independent Data Monitoring and Ethics Committee and responsible research ethics committee. No serious adverse events were reported.

Results

Feasibility evaluation

Recruitment took four months longer than planned due to delays in Site 2, resulting in a revised sample size of 48. Obtaining keyworker information on service users approached about trial participation proved impractical.

All referred service users (n=89, 100.0%) consented to research contact (see Figure 1). Adult mental health services (AMHS) referred 30 (33.7%) parents, child and adolescent mental health services (CAMHS) 29 (32.6%), and children's social care (CSS) 30 (33.7%). Site 1 referred sixty-five (73.0%) parents. Researchers made contact with 87 (97.7%) parents, requiring 1-13 communications per participant.

Sixty (69.7%) parents met initial criteria and completed screening. The most common reason for ineligibility was parents declining trial participation (n=12, 13.5%). Six parents were excluded due to child-related reasons, most commonly presence of child developmental disorder.

Forty-eight consenting participants met parent and child screening criteria, all of whom completed baseline measures, representing 80.0% of screened parents, 53.9% of referred

For peer review only - http://bmjopen.bmj.com/site/about/guidelines.xhtml

parents and 68.8% of the planned sample. Five (8.3%) met neither parent nor child screening criteria, four (6.7%) did not meet SAPAS criterion, and three (5.0%) did not meet SDQ criterion.

Thirty-six (75.0%) participants were from Site 1, exceeding the site recruitment target. Site 2 recruitment (n=12) was 34.3% of that planned, due to delayed recruitment and lower service engagement. Eighteen (37.5%) participants were referred by AMHS, 16 (33.3%) CAMHS and 14 (29.2%) CSS.

There was a significant difference in trial condition uptake (HFP-M: n=21, 87.5%; Usual care n=15, 62.5%; $\chi 2=4.0$, df=1 (48), $p<0.05$). Modal duration between randomisation and starting HFP-M was 2 weeks (range 1-23 weeks). Parents declined HFP-M because one gave birth, another was in couple conflict about participation, and a third did not respond to persistent contact (see Figure 1). The modal duration before Usual care parenting session receipt was 5.5 weeks (range 1-17 weeks). The most common reason for declining the additional Usual care parenting session was that participants hoped to be allocated to HFP-M.

Thirty-two (66.7%, 95% CI 51.6% to 79.6%) participants completed post-intervention measures. The majority of participants who did not take up the intervention offered, mainly in the Usual care condition, did not complete post-intervention measures.

Twenty-one (65.6%) parents completing post-intervention measures were also assessed at follow-up, representing 43.8% of all participants. Post-intervention measures were completed a mean of 42.0 weeks (SD 14.6) after baseline, due to HFP-M delivery being lengthier in duration than planned. Follow-up measures were completed a mean of 20 weeks (SD 8.9) later. Researchers required one to eight participant contacts to arrange post-intervention and follow-up data collection. Main reasons for non-completion included unable to contact participant, participant declining completion, and participant health and life circumstances.

There were less than 0.1% missing data items across clinical measures at each time point.

Sample characteristics

Demographic characteristics

All 48 participants were the biological parent of the index child, one was a father (2.1%) (see Table 1). The majority were lone parents (n=31, 67.4%), mean age, 34.9 (SD 7.1) years. The

For peer review only - http://bmjopen.bmj.com/site/about/guidelines.xhtml

majority (n=28, 61.0%) were White British/White, with fewer Black/Black British (n=9, 19.6%) and Dual Heritage (n=6, 13.5%) parents. English was the most common first language (n=44, 91.7%). Twenty-five (55.6%) participants completed education at 18 years or younger. Four participants (8.9%) were higher education graduates.

Most participants (n=37, 80.4%) were not in paid employment. Nine (19.5%) had partners in paid employment, predominantly part-time (n=7, 15.2%). No parent was in paid employment in 30 (65.2%) households. Twenty-five (86.2%) parents had received a formal psychiatric diagnosis, mean time since initial diagnosis was 9.7 (7.4) years.

Twenty-six (54.2%) children were male, mean age of 7.8 (SD 2.2) years. Median number of children in the home was 2, range 1-5 (see Table 1). Participants reported significant difficulties with anxiety/depression, pain/discomfort, with a smaller proportion experiencing difficulties in undertaking everyday activities (see Table 1).

Clinical characteristics

Over eighty per cent of ECBI Problem and Intensity scores at baseline exceeded the clinical caseness cut-off, with similar caseness rates for CBCL-Int and PS (see Table 2). The most common baseline CAMC child-related parent concerns were conduct, self-regulation, parent-child relationships and emotional distress (see Table 3).

Trial interventions acceptability

HFP-M attendance

Of 21 participants who accepted HFP-M, 13 (61.9%, 95% CI 38.4% to 81.9%) completed HFP-M within the trial period. Six (28.6%, 95% CI 11.3% to 52.2%) withdrew before completion due to acute adult mental health crisis and complex family circumstances unrelated to HFP-M receipt. Recruitment delay and longer than anticipated intervention duration resulted in two (9.5%, 95% CI. 1.2% to 30.4%) participants not fully completing HFP-M before trial conclusion. The mean number of HFP-M appointments offered was 15.8 (SD 7.7) and mean number attended was 11.2 (SD 6.3). HFP-M appointment attendance was 70.2%. Mean duration of HFP-M delivery was 28.4 (SD 21.7) weeks.

HFP-M therapeutic alliance acceptability

Eighteen (85.3%) HFP-M participants completed post-intervention WAI-SR (mean total score=73.8, SD 10.4). Mean subscale scores were consistently in the upper end of the scale

For peer review only - http://bmjopen.bmj.com/site/about/guidelines.xhtml

(Mean Tasks Subscale score=24.9, SD 3.5; Bond Subscale score=24.9, SD 3.9; Goals Subscale score=23.9, SD 4.1).

Usual Care

All 15 participants accepting the additional parenting session completed. Due to lower retention, only six participants provided post-intervention WAI-SR data (mean total score=56.2, SD 18.8). Mean subscales scores were consistently lower than the HFP-M condition (Mean Tasks Subscale score=17.4, SD 7.2; Bond Subscale score=19.8, SD 7.1; Goals Subscale score=19.0, SD 7.6).

There appeared to be a substantial difference in WAI-SR scores between the two conditions but there was insufficient Usual care data to test for a statistical difference.

No adverse events were reported during the trial. Participant intervention withdrawal most frequently occurred due to deterioration in participant mental health and life circumstances, unrelated to trial participation.

Clinical outcomes

There were estimated mean improvements from baseline scores across a number of outcomes within both trial conditions (see Table 4). HFP-M mean differences exceeded those in Usual care on several outcomes. These findings should be treated with caution given the wide confidence intervals.

Estimated effect sizes showed a general post-intervention advantage for HFP-M on a range of outcomes. Medium effects for child behavioural problem severity (ECBI Intensity, ES 0.4, CI -0.3-1.1), and parenting satisfaction (KPSS, ES 0.4, CI -0.3-1.1) were detected and a large effect for parent-reported reductions in concerns about their child (CAMC Problem 1, ES 1.2, CI 0.4-2.0, CAMC Problem 2, ES 1.3, CI 0.5-2.1). No effects were detected for parenting behaviour and adult mental health. Descriptive scrutiny of follow-up findings showed that outcome scores across both groups were generally maintained or continued to improve.

Estimates of standard deviation and upper confidence intervals (u80% CI) for future power calculations of main clinical outcomes are: ECBI Problem: SD 6.7 (u80%CI: 7.6), ECBI Intensity SD 33.6 (u80%CI: 37.1), CAMC Problem 1: SD 15.3 (u80%CI: 17.4), and KPSS: 3.0 (u80%CI: 3.3).

For peer review only - http://bmjopen.bmj.com/site/about/guidelines.xhtml

Health economic findings

Details of the health economic analyses are provided in full in Day et al. At Time 2, CSRI data were available for 26 cases and 19 at Time 3. CSRI Time 2 data revealed that the services most used by caregivers included GPs, psychiatrists, other medics and social workers. Caregivers were often in receipt of medication. Children were frequently in contact with school nurses, dentists, opticians, and GPs. EQ-5D-5L data were available for 36 caregivers at Time 1, 32 at Time 2 and 21 at Time 3. EQ-5D-Y data were available for 38, 31 and 20 children at each of the respective time points.

Conclusion

This study assessed feasibility, potential challenges and decision-making for a definitive HFP-M trial.[28-30,39] Participant identification methods were successful across a wide range of mental health and CSS teams, over 90% of referred parents met dual child and parent screening criteria. Non-diagnostic eligibility criteria were acceptable and effective in recruiting an appropriate, multi-morbid sample.[61,62] Negative life events and disrupted family functioning, characteristic of the sample population, commonly delayed screening, data collection and intervention delivery.[7] Nevertheless, participant retention exceeded the *a priori* primary feasibility criterion of 65%. Participants declining intervention conditions were less likely to be retained at follow-up. Participant recruitment was slower than planned, mainly due to operational difficulties in one site.

Participants' multi-morbid characteristics are commonly associated with poorer treatment engagement and outcomes.[63,64] Intervention uptake differed substantially between trial conditions. HFP-M was largely acceptable to participants but delivery was less efficient than planned, often due to parents' life circumstances. The augmented Usual care condition appeared to be less acceptable, and potentially affected participant retention. Lower Usual care retention and acceptability rates may reflect common dissatisfaction associated with control condition allocation.[65,66]

Clinical effects were detected across trial conditions, with potential advantage for child behaviour, parental child concerns and parenting satisfaction for HFP-M. These are welcome given the population's complex parenting impairments and negative treatment expectancies.[2,64] The final sample size limited trial power and consequently affected

For peer review only

interpretation of results. A definitive trial could potentially narrow child selection criteria to include only behaviour problems as parenting programmes have a stronger evidence base for this condition. Alternatively, HFP-M content may require strengthening specifically in relation to child internalising difficulties. Economic findings indicated potential cost advantages for HFP-M over usual care.[44] However, CSRI completion rates were not high and a simpler version may be required in a larger trial.

Trial conditions may have differed in duration, location and therapeutic intensity, potentially accounting for outcome and acceptability differences. Data collection relied on parent self-report, which is conventional given poor reliability of child-report across the age group and costs associated with independent ratings. Trial therapists provided self-report intervention fidelity data and supervision examined therapist HFP-M skills and implementation. Independent methods could strengthen validity of fidelity monitoring in a definitive trial, including observational and video methods.

A definitive trial is potentially feasible and should be based on the assumption of a medium effect size for the primary outcome of child behaviour. Site engagement, resource allocation and keyworker training in participant identification and recruitment will be crucial to enrolment of the larger sample required in a future trial. Embedded researchers assisting in caseload identification and direct parent recruitment, not possible in this feasibility study, may promote enrolment. Participant retention, particularly parents allocated to a usual care condition, will continue to be challenging for a full trial. The population's complex personality difficulties and, typically, heightened sensitivity to rejection, underline the importance of managing sensitively and effectively trial consent procedures, the emotional and practical consequences of random allocation and proactively maintaining communication and validating relationships with participants throughout trial duration, particularly for those allocated to usual care. Though not routinely available, the usual care condition could be augmented with an on-going parent support group to potentially increase equipoise, face validity and uptake, which may also benefit trial retention.

HFP-M clinical and trial efficiency may be improved with more explicit, validating discussion with participants about the potential impact of life and personal circumstances on attendance, use of pre-emptive cancellation plans, and inclusion of inter-session contact using digital technology.[31,64,67]

For peer review only - http://bmjopen.bmj.com/site/about/guidelines.xhtml

Figure 1: CONSORT diagram randomised feasibility trial recruitment and retention

Word count: 4187

For peer review only

For peer review only - http://bmjopen.bmj.com/site/about/guidelines.xhtml

References

[1] Fergusson, D., Horwood, L. & Ridder, E. (2005). Show me the child at seven: The consequences of conduct problems in childhood for psychosocial functioning in adulthood. *J Child Psychol Psyc,* 46*, 837-849.*

[2] Petfield, L., Startup, H., Drosher, H. & Cartwright-Hatton, S. (2015) Parenting in mothers with borderline personality disorder and impact on child outcomes. *Evid Based Ment Health*, 18(3), 67-75.

[3] Eyden, J., Winsper, C., Wolke, D., Broome, M. & MacCallum, F. (2016). A systematic review of the parenting and outcomes experienced by offspring of mothers with borderline personality pathology: Potential mechanisms and clinical implications. *Clin Psychol Rev*, 47, 85–105.

[4] Day, C., Harris., L., McCrone, P., McMurran, M., Moran, P., Morgan, L.et al. (2017) Feasibility trial of a psychoeducational intervention for parents with personality difficulties: The Helping Families Programme. *Contemporary Clinical Trials Communications*, 8, 67-74. doi.org/10.1016/j.conctc.2017.08.002.

[5] Ackerson, B. (2003). Coping with the dual demands of severe mental illness and parenting: The parents' perspective. *Fam Soc*, 84, 109-119.

[6] Newton-Howes, G., Weaver, T. & Tyrer, P. (2008). Attitudes of staff towards patients with personality disorder in community mental health teams. *Aust NZ J P Psychiat*, *42,* 572-577.

[7] Coid, J., Yang, M., Tyrer, P. & Roberts, A. (2006). Prevalence and correlates of personality disorder in Great Britain. *Brit J Psychiat*, 188, 423-431.

[8] Newton-Howes, G., Tyrer, P., Anagnostakis, K., Cooper, S., Bowden-Jones, O. & Weaver, T. (2010). The prevalence of personality disorder, its comorbidity with mental state disorders, and its clinical significance in community mental health teams. *Soc Psych Psych Epid, 45,* 453-460.

[9] McCrone, P., Patel, A., Knapp, M. & Lawton-Smith, S. (2008). *Paying the price: The cost of mental health care in England to 2026*. London: King's Fund. Available at:

For peer review only

For peer review only - http://bmjopen.bmj.com/site/about/guidelines.xhtml

http://www.kingsfund.org.uk/sites/files/kf/Paying-the-Price-the-cost-of-mental-health-care-England-2026-McCrone-Dhanasiri-Patel-Knapp-Lawton-Smith-Kings-Fund-May-2008_0.pdf

[10] Johnson, J., Cohen, P., Kasen, S., Ehrensaft, M. & Crawford, T. (2006). Associations of parental personality disorders and axis I disorders with childrearing behavior. *J Am Acad Child Psy*, 69, 336-350.

[11] Newman, L., Stevenson, C., Bergman, L. & Boyce, P. (2007). Borderline personality disorder, mother-infant interaction and parenting perceptions: Preliminary findings. *Aust NZ J Psychiat*, 41, 598-605.

[12] Green, H., McGinnity, A., Meltzer, H., Ford, T. & Goodman, R. (2005). *Mental health of children and young people in Great Britain, 2004.* Basingstoke: Palgrave Macmillan. Available at: http://www.esds.ac.uk/doc/5269/mrdoc/pdf/5269technicalreport.pdf

[13] Davis, H., Day, C., Cox, A. & Cutler L. (2000). Child and adolescent mental health needs assessment and service implications in an inner-city area. *Clin Child Psychol P,* 5, 169-*188.*

[14] Romeo R, Knapp M. & Scott S. (2006). Economic cost of severe antisocial behaviour in children and who pays it. *Br J Psychiatry*, 188(6):547–553.

[15] Parsonage M, Khan L. & Saunders (2014). *Building a better future: The lifetime costs of childhood behavioural problems and the benefits of early intervention*. Lond Cent

Ment Health.

[16] Sved Williams, A., Yelland, C., Hollamby, S., Wigley, M. & Aylward, P. (2018) A new therapeutic group to help women with borderline personality disorder and their infants. *J Psychi Prac*, 24 (5): 331-340.

[17] Department of Health, Department for Education (2017). *Transforming Children and Young People's Mental Health Provision: A Green Paper.* London: DoH/DfE.

[18] Social Care Institute for Excellence (2011) Think child, think parent, think family: a guide to parental mental health and child welfare. https://www.scie.org.uk/publications/guides/guide30/summary.asp. Last accessed 31.05.18.

For peer review only - http://bmjopen.bmj.com/site/about/guidelines.xhtml

[19] Mental Health Taskforce (2016). The Five Year Forward View of Mental Health. Mental Health taskforce. Available at: www.england.nhs.uk/mentalhealth/taskforce. Last accessed 22.11.18

[20] Parker, G., Beresford, B., Clarke, S. et al (2008). *Technical report for SCIE Research Review on the prevalence and incidence of parental mental health problems and the detection, screening and reporting of parental mental health problems*. Working Paper No. SCIE 2247: University of York.

[21] Wilson, R., Weaver, T., Michelson, D. & Day, C. (2018) Experiences of parenting and clinical intervention for mothers affected by personality disorder: a pilot qualitative study combining parent and clinician perspectives. *BMC Psychiatry,* 18,152. doi.org/10.1186/s12888-018-1733-8.

[22] National Institute for Health and Clinical Excellence (2013). *Antisocial behaviour and conduct disorders in children and young people: Recognition, intervention and management.* London: NICE.

[23] Michelson, D., Day, C., Dretzke., Davenport, C. & Barlow, J. (2013) Do evidence-based interventions work when tested in the "real world?" A systematic review and meta-analysis of parent management training for the treatment of child disruptive behavior. *Clin Child Fam Psych,* 16(1), 18-34.

[24] Reyno, S. & McGrath, P. (2006). Predictors of parent training efficacy for child externalizing behavior problems: A meta-analytic review. *J Child Psychol Psyc*, 47, 99-111.

[25] Lundahl, B., Risser, H. J., & Lovejoy, M. C. (2006). A meta-analysis of parent training: Moderators and follow-up effects. *Clin Psychol Rev*, *26*(1), 86-104.

[26] Dawe, S., & Harnett, P. (2007). Improving family functioning in methadone maintained families: Results from a randomised controlled trial. *J Subst Abuse Treat,* 32, 381-390.

[27] Thanhäuser M, Lemmer G, de Girolamo G, & Christiansen H. (2017) Do preventive interventions for children of mentally ill parents work? Results of a systematic review and metanalysis. *Curr Opin Psychiatry*, 30(4):283–299.

[28] Craig, P., Dieppe, P., Macintyre, S., Michie, S., Nazareth, I. & Petticrew, M. (2008).

For peer review only - http://bmjopen.bmj.com/site/about/guidelines.xhtml

Developing and evaluating complex interventions: New guidance. London: Medical Research Council.

[29] Hoffman, T., Glasziou, P., Boutron,, I., Milne, R., & Perera, R. (2014). Better reporting of interventions: Template for intervention description and replication (TIDieR) checklist and guide. *Brit Med J*, 348:g1687

[30] Eldridge, S., Chan, C., Campbell, M., Bond, C., Hopewell, S., Thabane, L et al. (2016). CONSORT 2010 statement: extension to randomised pilot and feasibility trial *Pilot Feasibility Stud*, 2:64.

[31] Bateman, A. W., & Fonagy, P. (2000). Effectiveness of psychotherapeutic treatment of personality disorder. *Brit J Psychiat*, 177(2), 138-143.

[32] McMurran, M., Crawford, M. J., Reilly, J.G., McCrone, P., Moran, P., Williams, H., et al. (2011). Psycho-education with problem solving (PEPS) therapy for adults with personality disorder: A pragmatic multi-site community-based randomised clinical trial. *Trials*, 12, 198.

[33] Day, C., Kowalenko, S., Ellis, M., Dawe, S., Harnett, P. & Scott, S. (2011). The Helping Families Programme: A new parenting intervention for children with severe and persistent conduct problems. *Child Adol Ment H-UK*, 16, 167-171.

[34] Harnett, P. & Dawe, S. (2008). Reducing Child Abuse Potential in Families Identified by Social Services: Implications for Assessment and Treatment. *Brief Treatment and Crisis Intervention*, 8(3), 226–235.

[35] Gray, A. Townsend, M. Bourke, M. & Grenyer, B. (2018) Effectiveness of a brief parenting intervention for people with borderline personality disorder: a 12-month follow-up study of clinician implementation in practice, Advances in Mental Health, 17:1, 33-43, doi: 10.1080/18387357.2018.1464887

[36] Day, C., Ellis., M. & Harris. (2015). *Family Partnership Model Reflective Practice Handbook*. 2nd Edn. South London & Maudsley NHS Foundation Trust/King's College, London.

[37] McMurran, M., Huband, N. & Overton, E. (2010). Non-completion of personality disorder treatments: A systematic review of correlates, consequences, and interventions. *Clin Psychol Rev*, 30, 277-287

For peer review only - http://bmjopen.bmj.com/site/about/guidelines.xhtml

[38] Tetley, A., Jinks, M., Huband, N., Howells., K & McMurran, M. (2012). Barriers to and facilitators of treatment engagement for clients with personality disorder: A Delphi survey. *Personal Ment Health*, 6, 97-110.

[39] Kazdin AE. (1980) Acceptability of alternative treatments for deviant child behavior. *J Appl Behav Anal*., 13(2):259–273.

[40] Foster SL, Mash EJ. (1999) Assessing social validity in clinical treatment research: Issues and procedures. *J Consult Clin Psychol*., 67(3):308.

[41] Bugge, C., Williams, B., Hagen, S., Logan, J., Glazener, C., Pringle, S., et al. (2013). A process for Decision-making after Pilot and feasibility Trials (ADePT): development following a feasibility study of a complex intervention for pelvic organ prolapse. *Trials*, 14:353. doi: 10.1186/1745-6215-14-353. PubMed.

[42] Oud M, Arntz A, Hermens ML, Verhoef R, Kendall T. (2018) Specialized psychotherapies for adults with borderline personality disorder: A systematic review and meta-analysis. *Aust N Z J Psychiatry*.; 52(10):949–961.

[43] McMurran M, Huband N, Overton E. (2010) Noncompletion of personality disorder treatments: A systematic review of correlates, consequences, and interventions. *Clin Psychol Rev*., 30(3):277–287.

[44] Day C, Briskman J, Crawford MJ, Foote L, Harris L, Boadu J, et al. Helping Families Programme-Modified: Development of a Specialised Parenting Intervention for Parents Affected by Severe Personality Difficulties and Randomised Feasibility Trial. *Health Technol Assess*. *Accepted for publication*.

[45] Moran, P., Leese, M., Lee, T., Walters, P., Thornicroft, G. & Mann, A. (2003). The Standardised Assessment of Personality - Abbreviated Scale (SAPAS): Preliminary validation of a brief screen for personality disorder. *Brit J Psychiat*, 183, 228-232.

[46] Goodman, R., Ford, T., Richards, H., Gatward, R. & Meltzer, H. (2000). The Development and Well-Being Assessment: Description and initial validation of an integrated assessment of child and adolescent psychopathology. *J Child Psychol Psyc*, 41, 645-655.

For peer review only - http://bmjopen.bmj.com/site/about/guidelines.xhtml

[47] Day, C., Michelson, M., Thomson, S., Penney, C. & Draper, L. (2012). Evaluation of a peer-led parenting intervention for child behaviour problems: A community-based randomised controlled trial. *Brit Med J*, 344, e1107.

[48] van Reenen, M. & Janssen, B. (2015). *EQ-5D-5L User Guide*. Available at: https://euroqol.org/wp-content/uploads/2016/09/EQ-5D-5L_UserGuide_2015.pdf [Accessed 29 May 2018].

[49] Wille, N., Badia, X., Bonsel, G., Burstro, K., Cavrini, G., Develin, N., et al. (2010). Development of the EQ-5D-Y: A child-friendly version of the EQ-5D. Qual Life Res, 19 (6), 875-86.

[50] Eyberg, S. & Ross, A.W. (1978). Assessment of child behaviour problems: The validation of a new inventory. *J Clin Child Psychol*,7:2,113-116, *doi*:10.1080/15374417809532835

[51] Achenbach, T. & Rescorla, L. (2001). *Manual for the ASEBA School-Age Forms and Profiles*. Burlington, VT: University of Vermont, Research Center for Children, Youth & Families.

[52] Arnold, D., O'Leary, S., Wolff, L. & Acker, M. (1993). The Parenting Scale: A measure of dysfunctional parenting in discipline situations. *Psychol Assessment*, 5, 137-144.

[53] James, D., Schumm, W., Kennedy, C., Grigsby, C., Shectman, K. & Nichols, C. (1985). Characteristics of the Kansas parental satisfaction scale among two samples of married parents. *Psychol Rep*, 57, 163-169.

[54] Müller, J., Postert, C., Beyer, T., Furniss, T. & Achtergarde, S. (2010). Comparison of eleven short versions of the Symptom Checklist 90-Revised (SCL-90-R) for use in the assessment of general psychopathology. *Psychol Assessment*, 32, 246-254.

[55] Hatcher, R., & Gillaspy, J. (2006). Development and validation of a revised short version of the Working Alliance Inventory. *Psychother Res*, 16, 12-25.

[56] Beecham J, Knapp M. Costing psychiatric interventions. In Thornicroft G (ed) Measuring Mental Health Needs. London, Gaskell.

For peer review only

For peer review only - http://bmjopen.bmj.com/site/about/guidelines.xhtml

[57] Hertzog, M. (2009). Considerations in determining sample size for pilot studies. *Res Nurs Health*, 31, 180-191.

[58] Browne, R. (1995). On the use of a pilot sample for sample size determination. *Stat Med,* 14, 1933-1940.

[59] Brown, H. & Prescott. R. (2007). *Applied Mixed Models in Medicine. Second Edition.* New York: John Wiley & Sons.

[60] INVOLVE (2010). *Payment for Involvement: A guide for making payments to members of the public actively involved in NHS, public health and social care research*. INVOLVE: Eastleigh.

[61] McMurran, M., Delport, J., Wood, K., Jenkins, S., Wall, M. & Day, F. (2012). Recruitment to personality disorder treatment trials. *MHRJ*, 17, 119-128.

[62] Stines, L. & Feeny, N. (2008) Unique ethical concerns in clinical trials comparing psychosocial and psychopharmalogical interventions. *Ethics Behav*, 18:2-3, 234-246, doi: 10.1080/10508420802064333

[63] Cooper, A & Conklin, L (2015) Dropout from individual psychotherapy for major depression: A meta-analysis of randomized clinical trials. *Clin Psychol Rev*, 40 57–65

[64] Barnicot, K., Katsakou, C., Marougka, S. & Priebe, S. (2011) Treatment completion in psychotherapy for borderline personality disorder – a systematic review and meta-analysis. *Acta Psychiat Scand*, 123: 327–338

[65] Lindström, D., Sundberg-Petersson, I., Adami, J. & Tönnesen, H. (2010) Disappointment and drop-out rate after being allocated to control group in a smoking cessation trial. *Contemp Clin Trials*, 1, 22-6. doi: 10.1016/j.cct.2009.09.003.

[66] Cook T. & Campbell, D. (1979) Quasi-experimentation: Design and Analysis Issues for Field Settings. Chicago, Rand McNally.

[67] McMain, S., Boritz, T., & Leybman, M. (2015). Common strategies for cultivating a positive therapy relationship in the treatment of borderline personality disorder. *J Psychother Integr*, 25(1), 20-29.

For peer review only - http://bmjopen.bmj.com/site/about/guidelines.xhtml

Table 1: Participant demographic characteristics

Demographic characteristics		Baseline (Time 1)		
		Total	Intervention	Usual Care
Parent gender (Female) (n, %)		47 (97.9)	24 (100.0)	23 (95.8)
Parent age (yrs.)(mean, SD)		34.9 (7.1)	34.7 (7.5)	35.0 (6.9)
Received psychiatric diagnosis (n, %)		25 (86.2)	16 (88.9)	9 (81.8)
Psychiatric diagnosis duration (yrs.)(mean, SD)		9.7 (7.4)	8.6 (7.0)	10.9 (8.1)
Parent relationship to index child (biological parent) (n, %)		46 (100.0)	24 (100.0)	22 (100.0)
Index child gender (male) (n, %)		26 (55.3)	12 (50.0)	14 (60.9)
Index child age (mean, SD)		7.8 (2.2)	7.7 (2.0)	7.9 (2.2)
Number of children at home (median, range)		2 (1-5)	2 (1-5)	2 (1-5)
Not in paid employment (n, %)		37 (80.4)	19 (79.2)	18 (81.8)
Lone parent (n, %)		31 (67.4)	14 (58.3)	17 (77.3)
Partner in employment (n, %)		9 (19.5)	6 (25.0)	3 (12.5)
Parent education (n, %)	Graduate	4 (8.9)	2 (8.3)	2 (9.5)
	University not completed	3 (6.7)	3 (12.5)	0 (0.0)
	Other e.g. NVQ	13 (28.9)	6 (25.0)	7 (33.3)
	Left school 18yrs	9 (20.0)	4 (16.7)	5 (23.8)
	Left school 16 years	7 (15.6)	4 (16.7)	3 (14.3)
	Left school under 16	9 (20.0)	5 (20.9)	4 (19.0)
Ethnicity (n, %)	White UK/White other	28 (61.0)	13 (54.2)	15 (68.2)
	Black UK/African Caribbean	9 (19.6)	7 (29.2)	2 (9.1)
	Dual heritage	6 (13.0)	3 (12.5)	3 (13.6)
	Black UK/African	2 (4.3)	0 (0.0)	2 (9.1)
	Other	1 (2.2)	1 (4.2)	0 (0.0)
Parent health status[1] (n, %)	Mobility problems	9 (25.0)	4 (23.5)	5 (26.4)
	Problems in self-care washing & dressing	3 (8.3)	1 (5.9)	2 (10.5)
	Difficulties in undertaking usual activities	13 (36.1)	7 (41.1)	6 (31.6)
	Suffered pain/discomfort	17 (47.2)	6 (35.3)	11 (57.9)

For peer review only - http://bmjopen.bmj.com/site/about/guidelines.xhtml

[1] EQ-5D-5L health moderate/severe status

For peer review only

For peer review only - http://bmjopen.bmj.com/site/about/guidelines.xhtml

Table 2: Participant baseline clinical caseness

Measure	Baseline		
	Total	Intervention	Usual care
ECBI Problem Caseness (≥15) (n, %)	41 (89.1)	21 (87.5)	20 (90.9)
ECBI Intensity Caseness (≥131) (n, %)	39 (83.0)	19 (79.2)	20 (83.3)
CBCL-Int (t-score) Caseness (≥60) (n, %)	45 (95.8)	23 (95.8)	22 (95.7)
PS Caseness (≥3.2) (n, %)	35 (74.5)	18 (75.0)	17 (73.9)

Table 3: Parent-reported concerns about index child

Concern Category	Primary	Secondary	Tertiary	Total
Conduct problems[1] (n, %)	18 (38.3)	27 (58.7)	24 (53.3)	69 (50.0)
Parent-child relationship & communication (n, %)	16 (34.0)	2 (4.4)	4 (8.9)	22 (15.9)
Child self regulation[2] (n, %)	4 (8.5)	11 (23.9)	7 (15.6)	22 (15.9)
Emotional distress[3] (n, %)	8 (17.0)	4 (8.8)	7 (15.6)	19 (13.8)
Other[4] (n, %)	1 (2.1)	2 (4.4)	1 (2.2)	4 (2.9)
School (n, %)	0 (0.0)	0 (0.0)	2 (4.4)	2 (1.5)
Total (n, %)	47	46	45	138

[1] including anger, tantrums, defiance, non-compliance, aggression, running away & lying; [2]including overactivity, poor concentration, overeating, & wetting; [3]including low mood, anxiety, low self-esteem; [4]including risk behaviours

For peer review only

For peer review only - http://bmjopen.bmj.com/site/about/guidelines.xhtml

Measure		Group	Baseline Mean (SD) (n)	Post-intervention Mean (SD) (n)	Follow-up Mean (SD) (n)	Baseline/Post-intervention Estimated mean difference (CI)	p	Effect Size
CAMC	Problem 1	Intervention	84.6 (16.0) (n=24)	45.2 (27.2) (n=18)	58.2 (29.5) (n=13)	18.633 (-40.177 to 2.910)	.087	1.2 (0.4-2.0)
		Usual Care	85.9 (14.7) (n=22)	63.1 (31.1) (n=14)	53.3 (34.4) (n=8)			
	Problem 2	Intervention	85.0 (19.1) (n=24)	51.1 (31.9) (n=18)	56.4 (35.5) (n=13)	22.486 (-44.318 to -.654)	.044	1.3 (0.5-2.1)
		Usual Care	82.9 (15.7) (n=22)	72.5 (26.0) (n=14)	66.5 (35.5) (n=8)			
	Problem 3	Intervention	81.0 (17.0) (n=24)	54.4 (33.8) (n=17)	42.8 (35.4) (n=13)	2.909 (-28.349 to 22.530)	.816	0.0 (-0.7-0.7)
		Usual Care	78.9 (18.5) (n=22)	57.3 (34.1) (n=14)	39.6 (24.3) (n=8)			
ECBI	Problem Score	Intervention	22.1 (7.4) (n=24)	17.9 (8.5) (n=15)	15.3 (9.4) (n=13)	1.559 (-4.24=55 to 7.374)	.585	0.1 (-0.7-0.88)
		Usual Care	22.43 (6.2) (n=21)	18.0 (11.5) (n=14)	18.8 (12.3) (n=8)			

For peer review only - http://bmjopen.bmj.com/site/about/guidelines.xhtml

	Intensity Score	Intervention	168.2 (35.9) (n=24)	142.4 (39.3) (n=24)	131.7 (43.0) (n=18)	12.866 (-4.24=55 to 7.374)	.233	0.4 (-0.3-1.1)
		Usual Care	169.5 (31.8) (n=23)	155.7 (49.6) (n=14)	148.9 (58.4) (n=8)			
CBCL-Int (t-score)		Intervention	72.9 (9.7) (n=24)	69.6 (10.4) (n=18)	68.2 (8.3) (n=13)	1.853 (-9.023 to 5.317)	.601	0.2 (-0.5-0.9)
		Usual Care	70.9 (11.9) (n=23)	70.9 (9.3) (n=14)	69.6 (8.6) (n=8)			
KPSS		Intervention	10.1 (3.1) (n=24)	12.9 (3.5) (n=18)	14.9 (3.8) (n=13)	1.177 (-1.260 to 3.615)	.331	0.4 (-0.3-1.1)
		Usual Care	10.9 (2.8) (n=23)	12.4 (3.9) (n=14)	13.6 (3.9) (n=7)			
PS		Intervention	111.0 (19.6) (n=24)	108.7 (16.5) (n=18)	98.3 (27.2) (n=13)	.210 (-14.776 to - 15.196)	.977	0.0 (-0.7-0.7)
		Usual Care	113.5 (24.8) (n=23)	108.7 (28.9) (n=14)	98.1 (26.9) (n=7)			
SCL-27 Global Severity Index		Intervention	1.8 (1.1) (n=24	1.6 (0.8) (n=18)	1.9 (0.8) (n=13)	-.105 (-.599 to .389)	.666	-0.1 (-0.8-0.6)
		Usual Care	1.7 (0.8) (n=22)	1.7 (0.9) (n=14)	1.3 (1.0) (n=8)			

For peer review only - http://bmjopen.bmj.com/site/about/guidelines.xhtml

Table 4: Parent reported child, parenting and parent clinical outcomes (intention to treat analysis)

For peer review only

For peer review only - http://bmjopen.bmj.com/site/about/guidelines.xhtml

For peer review only

For peer review only - http://bmjopen.bmj.com/site/about/guidelines.xhtml

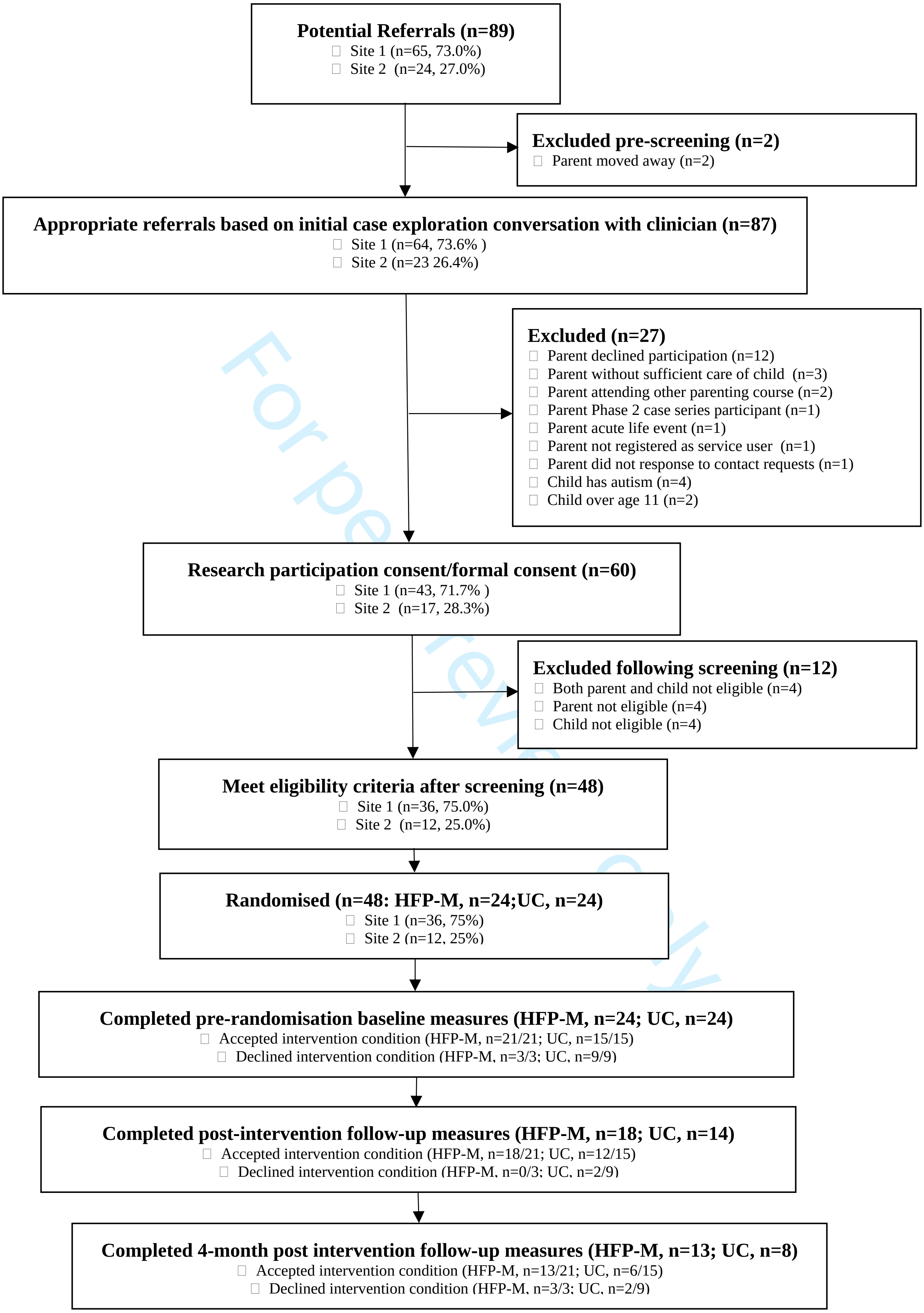

Figure 1: CONSORT diagram randomised feasibility trial recruitment and retention

For peer review only

For peer review only - http://bmjopen.bmj.com/site/about/guidelines.xhtml

CONSORT 2010 checklist of information to include when reporting a pilot or feasibility trial*

Section/Topic	Item No	Checklist item	Reported on page No
**Title and abstract**			
	1a	Identification as a pilot or feasibility randomised trial in the title	1
	1b	Structured summary of pilot trial design, methods, results, and conclusions (for specific guidance see CONSORT abstract extension for pilot trials)	2-3
**Introduction**			
Background and objectives	2a	Scientific background and explanation of rationale for future definitive trial, and reasons for randomised pilot trial	8-10
	2b	Specific objectives or research questions for pilot trial	10
**Methods**			
Trial design	3a	Description of pilot trial design (such as parallel, factorial) including allocation ratio	10
	3b	Important changes to methods after pilot trial commencement (such as eligibility criteria), with reasons	14, 15
Participants	4a	Eligibility criteria for participants	11
	4b	Settings and locations where the data were collected	13,14
	4c	How participants were identified and consented	13,14
Interventions	5	The interventions for each group with sufficient details to allow replication, including how and when they were actually administered	11,12
Outcomes	6a	Completely defined prespecified assessments or measurements to address each pilot trial objective specified in 2b, including how and when they were assessed	12,13
	6b	Any changes to pilot trial assessments or measurements after the pilot trial commenced, with reasons	N/A
	6c	If applicable, prespecified criteria used to judge whether, or how, to proceed with future definitive trial	10
Sample size	7a	Rationale for numbers in the pilot trial	13
	7b	When applicable, explanation of any interim analyses and stopping guidelines	N/A
Randomisation:			
Sequence generation	8a	Method used to generate the random allocation sequence	14
	8b	Type of randomisation(s); details of any restriction (such as blocking and block size)	10
Allocation concealment mechanism	9	Mechanism used to implement the random allocation sequence (such as sequentially numbered containers), describing any steps taken to conceal the sequence until interventions were assigned	14

For peer review only

Implementation	10	Who generated the random allocation sequence, who enrolled participants, and who assigned participants to interventions	14
Blinding	11a	If done, who was blinded after assignment to interventions (for example, participants, care providers, those assessing outcomes) and how	14
	11b	If relevant, description of the similarity of interventions	11,12
Statistical methods	12	Methods used to address each pilot trial objective whether qualitative or quantitative	14
**Results**			
Participant flow (a diagram is strongly recommended)	13a	For each group, the numbers of participants who were approached and/or assessed for eligibility, randomly assigned, received intended treatment, and were assessed for each objective	15,16,28
	13b	For each group, losses and exclusions after randomisation, together with reasons	15,16,28
Recruitment	14a	Dates defining the periods of recruitment and follow-up	14,15,16
	14b	Why the pilot trial ended or was stopped	N/A
Baseline data	15	A table showing baseline demographic and clinical characteristics for each group	29,30
Numbers analysed	16	For each objective, number of participants (denominator) included in each analysis. If relevant, these numbers should be by randomised group	15,16,28
Outcomes and estimation	17	For each objective, results including expressions of uncertainty (such as 95% confidence interval) for any estimates. If relevant, these results should be by randomised group	15,16,17,18, 19,31,32
Ancillary analyses	18	Results of any other analyses performed that could be used to inform the future definitive trial	N/A
Harms	19	All important harms or unintended effects in each group (for specific guidance see CONSORT for harms)	18
	19a	If relevant, other important unintended consequences	18
**Discussion**			
Limitations	20	Pilot trial limitations, addressing sources of potential bias and remaining uncertainty about feasibility	19,20
Generalisability	21	Generalisability (applicability) of pilot trial methods and findings to future definitive trial and other studies	20
Interpretation	22	Interpretation consistent with pilot trial objectives and findings, balancing potential benefits and harms, and considering other relevant evidence	19,20
	22a	Implications for progression from pilot to future definitive trial, including any proposed amendments	19,20
**Other information**			
Registration	23	Registration number for pilot trial and name of trial registry	ISRCTN1457 3230
Protocol	24	Where the pilot trial protocol can be accessed, if available	From Chief Investigator: rispin.1.day@ kcl.ac.uk

For peer review only

Funding	25	Sources of funding and other support (such as supply of drugs), role of funders	National Institute of Health Research, Health Technology Assessment, Project Reference Number: 12/194/01
	26	Ethical approval or approval by research review committee, confirmed with reference number	Health Research Authority South East Coast - Brighton & Sussex Research Ethics Committee (reference: 16/LO/0199)

Citation: Eldridge SM, Chan CL, Campbell MJ, Bond CM, Hopewell S, Thabane L, et al. CONSORT 2010 statement: extension to randomised pilot and feasibility trials. BMJ. 2016;355.

*We strongly recommend reading this statement in conjunction with the CONSORT 2010, extension to randomised pilot and feasibility trials, Explanation and Elaboration for important clarifications on all the items. If relevant, we also recommend reading CONSORT extensions for cluster randomised trials, non-inferiority and equivalence trials, non-pharmacological treatments, herbal interventions, and pragmatic trials. Additional extensions are forthcoming: for those and for up to date references relevant to this checklist, see www.consort-statement.org.

For peer review only
